# m6A‐related long noncoding RNAs predict prognosis and indicate therapeutic response in endometrial carcinoma

**DOI:** 10.1002/jcla.24813

**Published:** 2022-12-16

**Authors:** Xinying Zhou, Hu Zhang, Yingchun Duan, Jianlong Zhu, Haiyan Dai

**Affiliations:** ^1^ Department of Obstetrics and Gynecology Shanghai Pudong Hospital, Fudan University Pudong Medical Center Shanghai China

**Keywords:** bioinformatics analysis, endometrial cancer, immune infiltration, m6A‐associated lncRNA, prognosis

## Abstract

**Background:**

N6‐methyladenosine (m6A) has been identified as the most common, abundant, and conserved internal transcriptional modification. Long noncoding RNAs (lncRNAs) are noncoding RNAs consisting of more than 200 nucleotides, and the expression of various lncRNAs may affect cancer prognosis. The impact of m6A‐associated lncRNAs on uterine corpus endometrial carcinoma (UCEC) prognosis is unknown.

**Methods:**

In this study, UCEC prognosis‐related m6A lncRNAs were screened, bioinformatics analysis was performed, and experimental validation was conducted. Endometrial carcinoma (EC) and normal tissue samples were obtained from The Cancer Genome Atlas. The prognosis‐related m6A lncRNAs screened by the least absolute shrinkage and selection operator method were used for multivariate Cox proportional risk regression modeling. Principal component analysis and Gene Ontology, immune function difference, and drug sensitivity analyses of the prognostic models were performed. Prognostic analysis was conducted for m6A‐associated lncRNAs. The immune infiltration relationship of m6A‐associated lncRNAs in EC was identified using the ssGSEA immune infiltration algorithm. A competing endogenouse RNA network was constructed using the LncACTdb database. Finally, quantitative real‐time polymerase chain reaction (qRT‐PCR) assays were used to validate the differences in m6A‐related lncRNA expression in normal and EC cells.

**Results:**

CDKN2B‐AS1 and MIR924HG were found to be risk factors for EC. RAB11B‐AS1 was a protective factor in EC patients. MIR924HG expression was upregulated in KLE and RL95‐2 endometrial cancer cell lines. Prognostic models involved RAB11B‐AS1, LINC01812, HM13‐IT1, TPM1‐AS, SLC16A1‐AS1, LINC01936, and CDKN2B‐AS1. The high‐risk group was more sensitive to five compounds (ABT.263, ABT.888, AP.24534, ATRA, and AZD.0530) than the low‐risk group.

**Conclusion:**

These findings contribute to understanding of the function of m6A‐related lncRNAs in UCEC and provide promising therapeutic strategies for UCEC.

## Graphical abstract

Differential expression of m6A‐related lncRNA in normal and tumor tissues (by clinical stage and tumor grade) (ns, *p* ≥ 0.05; *p < 0.05; **p < 0.01; ***p < 0.001).

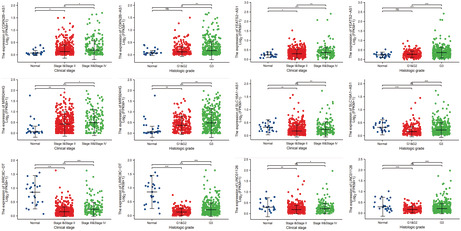

## 1 INTRODUCTION

Uterine corpus endometrial carcinoma (UCEC) is one of the three most common malignant tumors of the female reproductive system, accounting for 20%–30% of all malignant tumors of the female reproductive tract. In the recent years, the incidence of UCEC has started to increase and the age of those affected has become lower due to changes in the socioeconomic structure, changes in diet and lifestyle, and an increase in the number of people suffering from endocrine and metabolic diseases. Risk factors associated with endometrial carcinoma (EC) include persistent estrogen exposure, metabolic abnormalities (e.g., obesity and diabetes), early menarche, infertility, delayed menopause, EC genetic susceptibility genes (such as in Lynch syndrome), and advanced age. When diagnosed and confined to the uterus, 70% of EC cases are clinically identified early and have a good prognosis. The prognosis is poor in cases of advanced extrauterine metastasis and high‐risk histological types. FIGO staging of EC shows that the 5‐year survival rate exceeds 90% for stage I but drops to 70% for stage II and 60% for stage III.^1^ Currently, EC treatment is based on surgery and radiotherapy. The development of a treatment plan considers the patient's age, pathological type, molecular typing, clinical (imaging) staging, high‐risk factors, and physical status. As clinical research continues to develop, targeted therapy and immunotherapy have shown promising efficacy in advanced and recurrent metastatic EC in recent years. However, adjuvant treatment for UCEC remains complex and controversial, with a high risk of recurrence and death in advanced UCEC and few common treatment options for metastatic UCEC.^2^ Carbohydrate antigen 125 (CA125) helps in clinical outcome monitoring in the presence of ectopic lesions. However, CA125 can be abnormally increased by inflammation or radiation injury but may not be increased in some patients (e.g., isolated vaginal metastases). Therefore, CA125 is not an accurate predictor of recurrence without other clinical manifestations.^3^ Biomarkers and predictive models have been shown to improve targeted therapy and immunotherapy in oncology patients.^4^ Reliable biomarkers that reflect the prognosis and response to drug therapy have not been identified in UCEC.

N6‐methyladenosine (m6A) has been identified as the most common, abundant, and conserved internal transcriptional modification. The m6A modification is installed by m6A methyltransferases (METTL3/14, WTAP, RBM15/15B, and KIAA1429, called ‘writers’), reduced by demethylases (FTO and ALKBH5, called ‘erasers’) and recognized by m6A binding proteins (YTHDF1/2/3, IGF2BP1, and HNRNPA2B1, called ‘readers’).^5^ m6A RNA modifications regulate RNA transcription, splicing, processing, translation, and decay and are involved in the development and metastasis of various malignancies. It is well documented that m6A modifications can be a new biomarker for cancer diagnosis and prognosis. It has been reported that high METTL3 expression in primary hepatocellular carcinoma (HCC) increases the levels of m6A modification on the tumor suppressor SOCS2. YTHDF2 detects excessive chemical modification of m6SOCS2, accelerating SOCS2 degradation and ultimately leading to HCC.^6^ Ni et al.^7^ noted that high levels of MAGI3 m6A modification led to premature polyadenylation, shifting its functional role from oncogene suppression to dominant inactivation of oncogenes and promoting breast carcinogenesis.

Recent studies have shown that m6A modifications can regulate immune cell activation and tumor microenvironment infiltration, which may affect immunotherapy efficacy. Therefore, m6A modification is a potential target in cancer immunotherapy and is expected to complement immune checkpoint inhibitor therapy, thereby significantly improving cancer patient survival and quality of life.^8^, ^9^ The m6A writer METTL3 and reader YTHDF2 regulate the antitumor immunity of NK cells. METTL3‐mediated m6A methylation and YTHDF2 may be important regulators of antitumor immunity and NK cell homeostasis.^10^ Han et al. showed that the m6A reader YTHDF1 negatively regulates the antitumor immune response of dendritic cells (DCs). Specifically, YTHDF1 enhances the mRNA translation of a lysosomal protease that degrades antigens in the lysosome. Without YTHDF1, lysosomal protease translation is reduced, facilitating antigen cross‐presentation and promoting cytotoxic T‐cell responses.^11^

Additionally, numerous studies have shown that m6A modification regulates tumorigenesis and progression by modulating tumor metabolism and related signaling pathways. In colorectal cancer, overexpression of the m6A reader IMP2 (IGF2BP2) stabilizes the ZFAS1/OLA1 axis, thereby increasing OLA1 recruitment, ATP hydrolysis, and glycolysis; activating the Warburg effect; and enhancing cancer cell proliferation and colony formation.^12^ A hallmark of highly aggressive cancer cells is increased energy metabolism, including glycolytic activity and lactic acid fermentation. The Warburg effect is a typical feature of abnormal tumor glycolysis. In esophageal cancer, the m6A reader HNRNPA2B1 upregulates the fatty acid metabolism‐related genes ACLY and ACC1, thereby promoting cellular lipid accumulation and tumor progression, including cancer cell proliferation, migration, and invasion.^13^ Zhang et al.^14^ indicated that METTL3 can directly activate the PI3K‐Akt‐mTOR signaling pathway to promote cell proliferation, migration, and expression in retinoblastoma cancer cells.

Noncoding RNAs (ncRNAs) do not code for proteins but can produce noncoding transcripts that regulate gene expression, protein function, development, differentiation, and metabolism during physiological and pathological processes. Studies have shown that approximately 90% of the genes in eukaryotic genomes are transcribed genes. However, only 1%–2% of these transcribed genes encode proteins; most are transcribed as ncRNAs.^15^ ncRNAs are cancer pathway regulators identified in the last decade and are cancer biomarkers. Long noncoding RNAs (lncRNAs) are ncRNAs consisting of more than 200 nucleotides, and lncRNA expression may affect cancer prognosis. m6A regulators, such as METTL3, ALKBH5, and IGF2BP1, have been reported to m6A‐dependently modify ncRNAs involved in oncogenic effects.

Furthermore, ncRNAs can target and regulate m6A regulators, impacting cancer development.^16^ It has been shown that m6A‐modified lncRNAs can affect target gene function in tumors through RNA–protein binding, ceRNA (competitive endogenous RNA) mechanisms, or RNA–RNA binding. Many publications have reported a relationship between m6A modification and endometrial carcinogenesis and development. Liu et al. showed that METTL14 mutations or reduced METTL3 expression activates the AKT pathway, leading to increased proliferation and tumorigenicity of endometrial cancer cells. The above findings reveal that reduced m6A modification is an oncogenic mechanism in EC, confirming that m6A is an AKT signaling regulator.^17^ Zhang et al. found that IGF2BP1 is highly expressed in EC and associated with poor patient prognosis. Mechanistically, IGF2BP1 recognizes and stabilizes PEG10 mRNA in an m6A‐dependent manner, enhancing PEG10 expression and thereby accelerating the cell cycle and promoting EC progression.^18^ However, few studies have examined the association between m6A‐related lncRNAs and UCEC prognosis and treatment response.

This study aimed to analyze prognosis‐associated m6A lncRNAs in EC and construct a prognostic model that can predict overall survival (OS) and response to drug treatment in UCEC. Additionally, significant m6A‐related lncRNAs were individually analyzed and experimentally validated. The relationship between m6A related lncRNA and prognosis of EC was identified, and their expression in normal tissues and tumors was evaluated. Finally, immunoinfiltration analysis of m6A‐associated lncRNAs, mutation status, methylation degree analysis, ceRNA network construction of m6A‐associated lncRNA molecules, and Kyoto Encyclopedia of Genes and Genomes (KEGG) analysis were performed. In this study, we evaluated prognosis‐related m6A lncRNAs using public databases to identify potential EC treatment goals.

## 2 MATERIALS AND METHODS

### 2.1 Data acquisition

Transcriptome data (552 EC and 23 normal tissue samples) were downloaded from The Cancer Genome Atlas (TCGA) database.^19^ Perl software was used to differentiate between mRNA and lncRNA expression in EC. The m6A‐related gene expression was extracted using the “limma” package.^20^ The m6A‐associated lncRNAs were identified with absolute values of correlation coefficients >0.4 and *p* values <0.001. Coexpression analysis of m6A‐associated mRNAs and lncRNAs was performed using the “limma” package to obtain coexpression results. The “ggplot2” and “ggalluvial” packages were used to plot the coexpression profile in a Sankey map.

### 2.2 Identification of prognosis‐associated m6A lncRNAs

EC clinical data were downloaded from TCGA database, and m6A‐related lncRNA expression data were merged with EC survival data using the “limma” package. The prognosis‐associated m6A lncRNAs were obtained at *p* values <0.05 using the “survival” package. The “pheatmap”, “reshape2”, and “ggpubr” packages were used to draw forest, heat, and box line maps of prognosis‐related m6A lncRNAs.

### 2.3 Construction and validation of an m6A‐associated lncRNA prognostic model

EC samples from TCGA database were divided into test and training groups. The training group data were used to construct a prognostic model and obtain the formula for the prognostic model. Then, the test group data were used to verify the accuracy of the constructed model. The prognosis‐related m6A lncRNAs screened using the least absolute shrinkage and selection operator (LASSO) method were used for multivariate Cox proportional risk regression model construction. The risk scores were calculated using the following formula:

$$\text{Risk score}=\sum \left(\text{gene expression}\times \text{corresponding regression coefficient}\right).$$

The risk score for each sample can be obtained using the model formula. Based on the median value of the risk score, the patients were classified into two groups: high and low risk. Patients with a risk score above the median were classified as high risk, while those with a risk score below the median were classified as low risk. The R software packages “survival”, “caret”, “glmnet”, “survminer” and “timeROC” were used, with *p* < 0.05 determined by the Cox method as the criterion.

The constructed prognostic models were validated. The “limma”, “tidyverse”, “ggplot2”, and “ggExtra” packages were used to plot the correlation heatmap of m6A‐related lncRNAs and mRNAs. The “survivor” and “survminer” packages were used to plot the survival curves of the training and test groups. The “pheatmap” package was used to plot the risk curves of the training and test groups. Receiver operating characteristic (ROC) diagnostic curves were plotted for the prognostic model to measure the model's accuracy,^21^ including time‐dependent ROC curves and clinically relevant ROC curves. The “survival”, “survminer”, and “timeROC” packages were used to plot ROC curves. Principal component analysis (PCA)^22^ was used to detect differences in the m6A genes, m6A‐associated lncRNAs, and m6A‐associated lncRNA‐constructed risk models between the low‐ and high‐risk groups. The spatial distribution of samples is displayed in 3D scatter plots. The “limma” and “scatterplot3d” packages were used to construct the PCA.

### 2.4 Gene Ontology analysis of differentially expressed genes in high‐ and low‐risk groups in prognostic models, analysis of differences in immune function, and analysis of drug sensitivity

The “limma” package was used to obtain differentially expressed genes between the high‐ and low‐risk groups, followed by Gene Ontology (GO) analysis of the differentially expressed genes (BP, CC, MF) using the “clusterProfiler”, “org.Hs.eg.db” and “GOplot” packages. The “org.Hs.eg.db”, “GOplot”, “ggplot2” and “GOplot” packages were used to plot the circles and chord plots for the GO analysis. Differences in immune function between the high‐ and low‐risk groups were analyzed in the prognostic model. The relationship between the prognostic model and tumor immunity was investigated. The “limma”, “GSVA”, “GSEABase”, “pheatmap”, and “reshape2” packages were used to perform differential analysis of immune function in prognostic models. Drug sensitivity analysis was performed in the constructed prognostic models to predict the potential drugs that could be used to treat UCEC. IC50 values were used to evaluate the drug sensitivity in the prognostic model. The IC50 value can be used to measure the ability of a drug to induce apoptosis. The stronger the induction is, the lower the value and the more influential the treatment. The “limma”, “ggpubr”, “pRRophetic”, and “ggplot2” packages were used for drug sensitivity analysis of prognostic models.

### 2.5 Survival curves for m6A‐associated lncRNAs, univariate and multivariate analyses, and nomogram construction

Six lncRNAs from 32 prognosis‐associated m6A lncRNAs were selected, and the “survminer” and “survivor” packages were used to plot the prognosis‐associated m6A lncRNA survival curves. Then, a nomogram was constructed using the “rms” and “survivor” packages. Line plots were designed to assess the predictive effect of the risk scores obtained on the patient OS at 1, 3, and 5 years. Univariate and multivariate analyses of m6A‐related lncRNAs were performed using the “survival” and “ggplot2 packages to investigate the effect of m6A‐related lncRNAs on the prognosis of UCEC patients. RNAseq data in level 3 HTSeq‐FPKM format from TCGA (https://portal.gdc.cancer.gov/) UCEC (endometrial cancer) project with prognosis type OS were used.

### 2.6 Clinical features of EC associated with m6A‐related lncRNAs and differences in the expression of m6A‐related lncRNAs in normal and tumor tissues

A chi‐square independence test was used to investigate the relationship between m6A‐related lncRNAs and EC clinical stage, age, histological type, tumor grade, and OS. The differences in m6A‐related lncRNA expression in normal and tumor tissues were mapped using the “ggplot2” package at different clinical stages and tumor grades. RNAseq data in level 3 HTSeq‐FPKM format from TCGA (https://portal.gdc.cancer.gov/) UCEC (endometrial cancer) project were used. We also used the Gene Expression Profiling Interactive Analysis (GEPIA) database (http://gepia.cancer‐pku.cn/) to explore the expression of m6A‐related lncRNAs in different tumors and normal tissues.

### 2.7 Immune infiltration of m6A‐related lncRNAs in EC, intermolecular relationships, and mutations

Lollipop plots were plotted using the ssGSEA immune infiltration algorithm “GSVA” package in R software. Using a Spearman correlation test, the “ggplot2” package was applied to construct scatter plots to explore the intermolecular relationships of six m6A‐related lncRNAs in EC. We also used the GEPIA database for molecular correlation analysis. cBioPortal database^23^ (http://www. cbioportal.org/) is a visual tool to analyze cancer gene data, and the database was used for mutation, copy number, and m6A‐related lncRNA expression analysis of all UCEC samples.

### 2.8 Analysis of the methylation level of m6A‐associated lncRNAs in EC, ceRNA network construction, and GO and KEGG analysis of lncRNA‐associated ceRNAs

Scatter plots were constructed using the “ggplot2” package, and Spearman's correlation test was used to investigate m6A‐related lncRNA methylation in EC. Data were obtained using RNAseq data in level 3 HTSeq‐FPKM format from TCGA UCEC (Endometrial Cancer) project and Illumina human methylation 450 methylation data. GO and KEGG analyses of m6A lncRNA‐associated ceRNAs were performed using the LncACT‐Function project in the LncACTdb database (http://bio‐bigdata.hrbmu.edu.cn/LncACTdb/index.html) to determine the lncRNA‐associated dysregulations in ceRNA functions. The Uterus project in the LncACTdb database was used to find m6A lncRNA‐associated ceRNAs, and Cytoscape software^24^ was used to construct ceRNA networks.

### 2.9 Cancer Cell Line Encyclopedia database analysis and qRT‐PCR validation of m6A‐related lncRNAs

The m6A‐related lncRNA expression levels in different endometrial cancer cell lines were obtained from the Cancer Cell Line Encyclopedia (CCLE) database for bioinformatics analysis. Human UCEC (RL95‐2, Ishikawa, and KLE) cells and normal endometrial epithelial cells (NEECs) were obtained from Shanghai Yaji Biotechnology Company, Limited. All cell lines were cultured with the corresponding minimum essential medium containing 10% fetal bovine serum and 1% antibiotics in a CO2 incubator at 37°C. Immortalized endometrial epithelial cells were cultured using a medium specifically for endometrial cell immortalization. RL95‐2 cells were cultured using complete DMEM/F12 (containing 0.005 mg/ml insulin). Ishikawa cells were cultured in high‐sugar complete DMEM. KLE cells were cultured using complete DMEM/F12. Cells were lysed using TRIzol, cellular RNA was extracted, and RNA concentration and purity were determined using a NanoDrop® ND‐1000 spectrophotometer. Total RNA was reverse transcribed for cDNA synthesis using a Gene Amp PCR System 9700 (Applied Biosystems) according to the manufacturer's instructions. Relative lncRNA expression levels were determined by qRTPCR using the QuantStudio™ 5 Real‐time PCR System (Applied Biosystems). GAPDH was used as an internal control. The relative standard curve method was applied to estimate the relative expression of each lncRNA. Primer sequences are shown in Table S5.

### 2.10 Statistical analysis

All statistical analyses were performed using R software (v 4.1.2) and Perl software. The Kaplan–Meier method and univariate and multivariate Cox proportional risk regression models were used to analyze m6A‐associated prognostic lncRNAs. m6A‐associated lncRNAs and the clinical features of EC were explored using statistical methods, including a chi‐square test. The m6A‐associated lncRNA expression levels in normal cells/tissues and tumors were compared using a Kruskal–Wallis test. Spearman correlation analysis was used to identify intermolecular relationships between m6A‐related lncRNAs and the methylation degree in EC. A *p* value <0.05 was considered to indicate a statistically significant difference.

## 3 RESULTS

### 3.1 Screening and gene correlation analysis of m6A‐associated lncRNAs in EC

**FIGURE 1:**
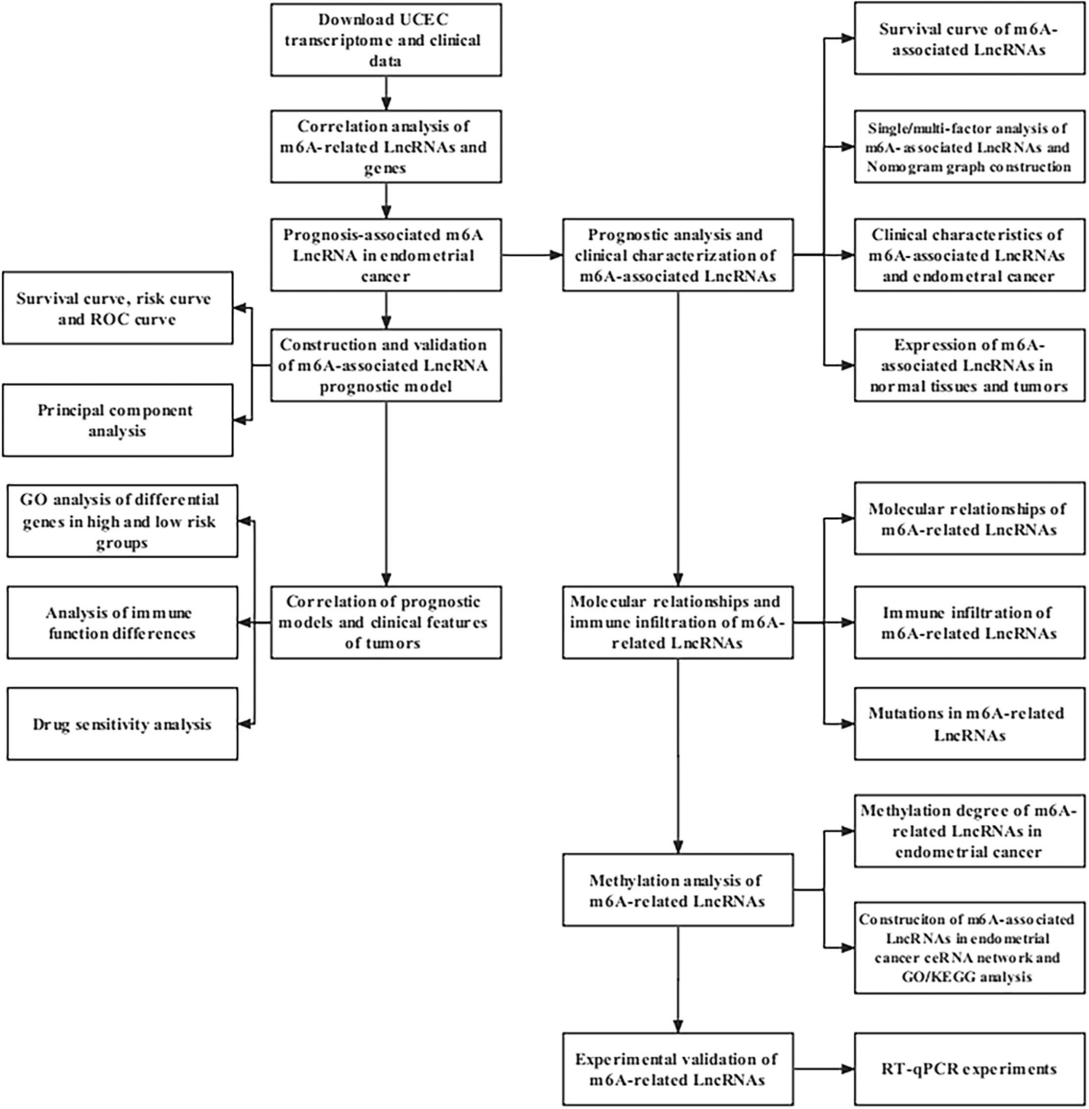
Detailed flow chart of the whole research

**FIGURE 2:**
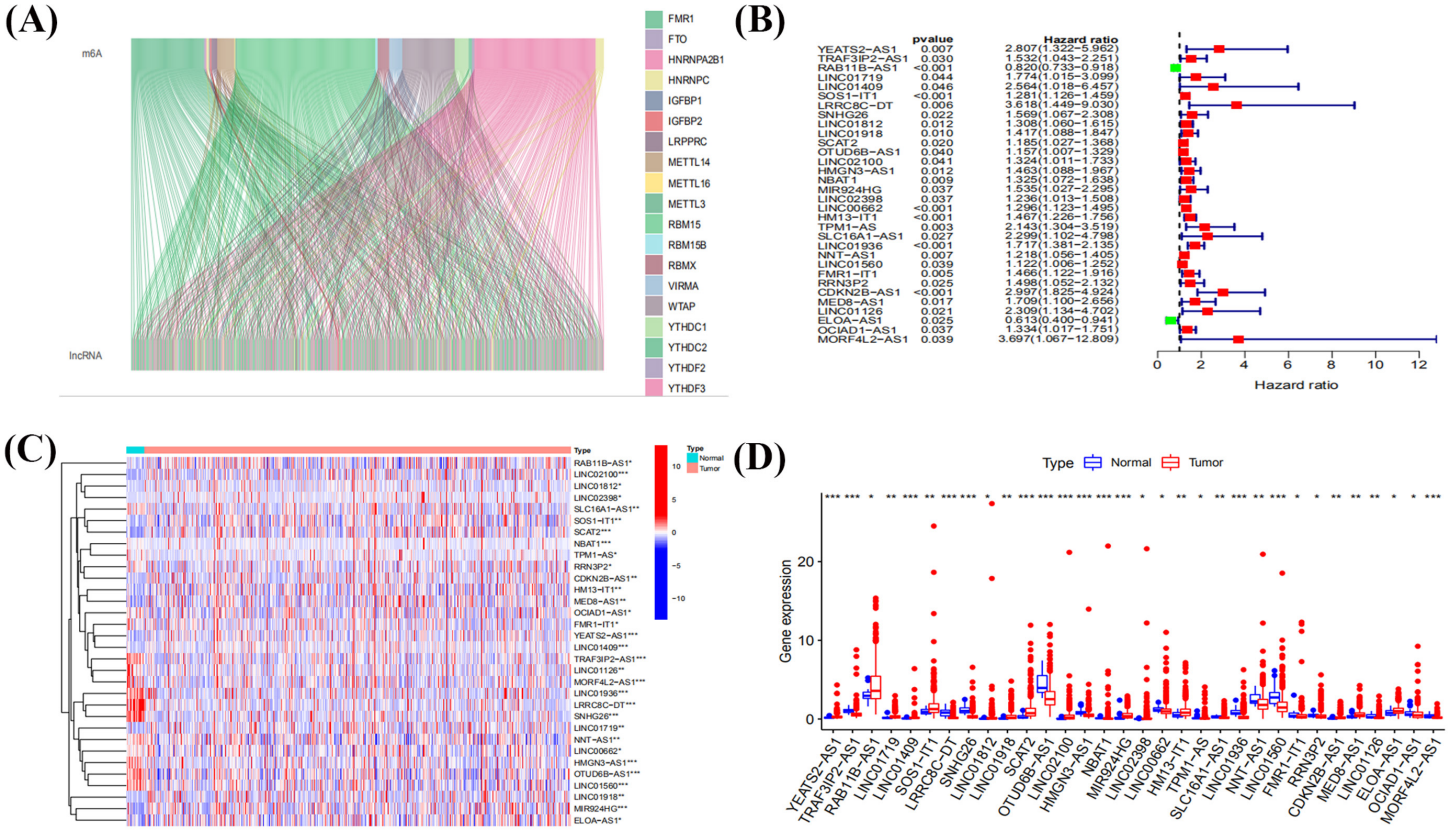
Analysis of m6A‐related mRNA and lncRNA co‐expression and prognostic molecular identification. (A) Sankey diagram of m6A‐associated mRNA and LncRNA co‐expression. (B) Forest plot of m6A prognosis‐associated lncRNAs. (C) Heat map of m6A prognosis‐associated lncRNA. (D) Box line plot of m6A prognosis‐associated lncRNAs (ns, *p* ≥ 0.05; *, *p* < 0.05; **, *p* < 0.01; ***, *p* < 0.001)

Figure 1 shows a detailed flow chart of our study, which includes prognostic model construction, m6A‐related lncRNA bioinformatics analysis, and experimental validation. Figure 2A shows that m6A‐related mRNAs (including the METTL3 methylation writer, the YTHDC1 methylation reader, and the FTO methylation eraser) are correlated with many m6A‐related lncRNAs in EC.

### 3.2 Identification of prognostic m6A‐associated lncRNAs in EC

Figure 2B shows that YEATS2‐AS1 (risk ratio, HR = 2.807; 95% confidence interval, 95% CI = 1.322–5.962; *p* = 0.007), CDKN2B‐AS1 (risk ratio, HR = 2.997; 95% confidence interval, 95% CI = 1.825–4.924; *p* < 0.001), and MIR924HG (hazard ratio, HR = 1.535; 95% confidence interval, 95% CI = 1.027–2.295; *p* = 0.037) are risk factors for EC. RAB11B‐AS1 (risk ratio, HR = 0.820; 95% confidence interval, 95% CI = 0.733–0.918; *p* < 0.001) is a protective factor for patients with EC. Figure 2C,D show m6A‐related lncRNA expression in normal and EC tissues. YEATS2‐AS1, CDKN2B‐AS1, and MIR924HG were all highly expressed in tumor tissues.

### 3.3 Construction and validation of an m6A‐associated lncRNA prognostic model

**FIGURE 3:**
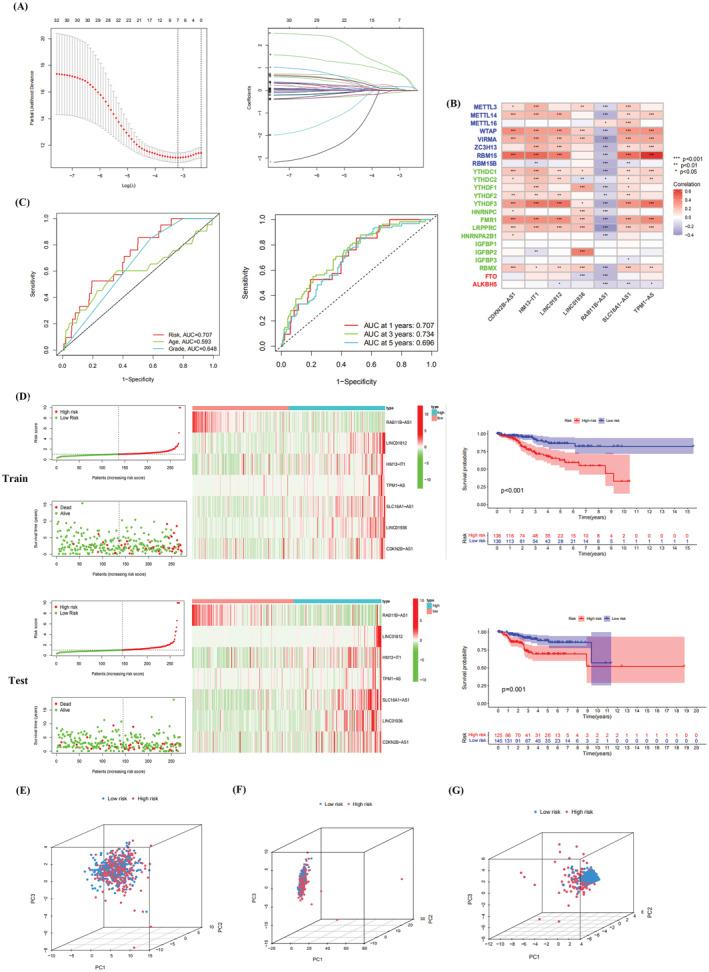
Construction, and validation of m6A‐associated lncRNA prognostic models. (A) LASSO regression model of m6A prognosis‐associated lncRNAs. (B) Correlation heat map of m6A‐associated lncRNA and mRNA (*p < 0.05; **p < 0.01; ***p < 0.001). (C) ROC diagnostic curves of prognostic models. (D) Risk curves, survival status plots, and risk heat maps of the prognostic models. Survival curves of the m6A‐associated lncRNA prognostic model. (E) PCA of the m6A gene. (F) PCA of m6A‐associated lncRNAs. (G) PCA of m6A‐associated lncRNAs in the prognostic model

The LASSO method was used to screen the prognosis‐associated m6A lncRNAs to construct multivariate proportional risk regression models (Figure 3A). We screened seven prognosis‐related m6A IncRNAs for the construction of prognostic models. We obtained risk scores via multivariate Cox regression with the following formula:

$$\begin{array}{cc} \text{Risk rating}= & \mathrm{RAB}1\text{1B}-\text{AS1}\times \left(-0.0953\right)+\text{LINC}01812\times \left(0.7762\right)\\ & +\mathrm{HM}13-\text{IT1}\times \left(0.0676\right)+\mathrm{TPM}1-\mathrm{AS}\times \left(0.1695\right)\\ & +\mathrm{SLC}1\text{6A}1-\text{AS1}\times \left(0.9066\right)+\text{LINC}01936\\ & \times \left(0.1754\right)+\text{CDKN}2B-\text{AS1}\times \left(0.0917\right). \end{array}$$

Then, we explored the correlation of the seven lncRNAs involved in the model construction with m6A genes (including m6A‐modified writers, readers, and erasers) (Figure 3B). cdKN2B‐AS1 was positively correlated with WTAP, RBM15, and YTHDF3 (*p* < 0.001). rAB11B‐AS1 was negatively correlated to VIRMA, RBM15B, and LRPPRC (*p* < 0.001). Figure 3C demonstrates that survival was significantly lower in the high‐risk group than in the low‐risk group in the training and test groups (*p* < 0.001). We calculated a risk score for each patient in the sample to assess the prognostic power of the model. Figure 3D depicts the distribution of m6A‐related lncRNAs and their expression levels. We can conclude that UCEC patients with higher risk scores had poorer OS than those with lower risk scores. rAB11B‐AS1 was expressed at low levels in the high‐risk group and at high levels in the low‐risk group, suggesting that it is a protective factor for UCEC prognosis. The remaining m6A‐related lncRNAs involved in the model construction were highly expressed in the high‐risk group and were risk factors for UCEC prognosis. The areas under the 1‐, 3‐, and 5‐year ROC curves were 0.707, 0.734, and 0.696, respectively (Figure 3E). The AUC of our constructed prognostic model had stronger predictive power than other clinicopathological characteristics (area under the ROC curve of 0.593 for age and 0.648 for tumor grade). Finally, we compared the risk models constructed with the m6A genes, m6A‐associated lncRNAs, and m6A‐associated lncRNAs differentially expressed between the low‐risk and high‐risk groups using PCA (Figure 3F–H). The results showed that our constructed m6A‐associated lncRNA prognostic model had the best ability to distinguish between the low‐ and high‐risk groups.

### 3.4 GO analysis of differentially expressed genes in the high‐ and low‐risk groups in prognostic models, analysis of differences in immune function, and analysis of drug sensitivity

**FIGURE 4:**
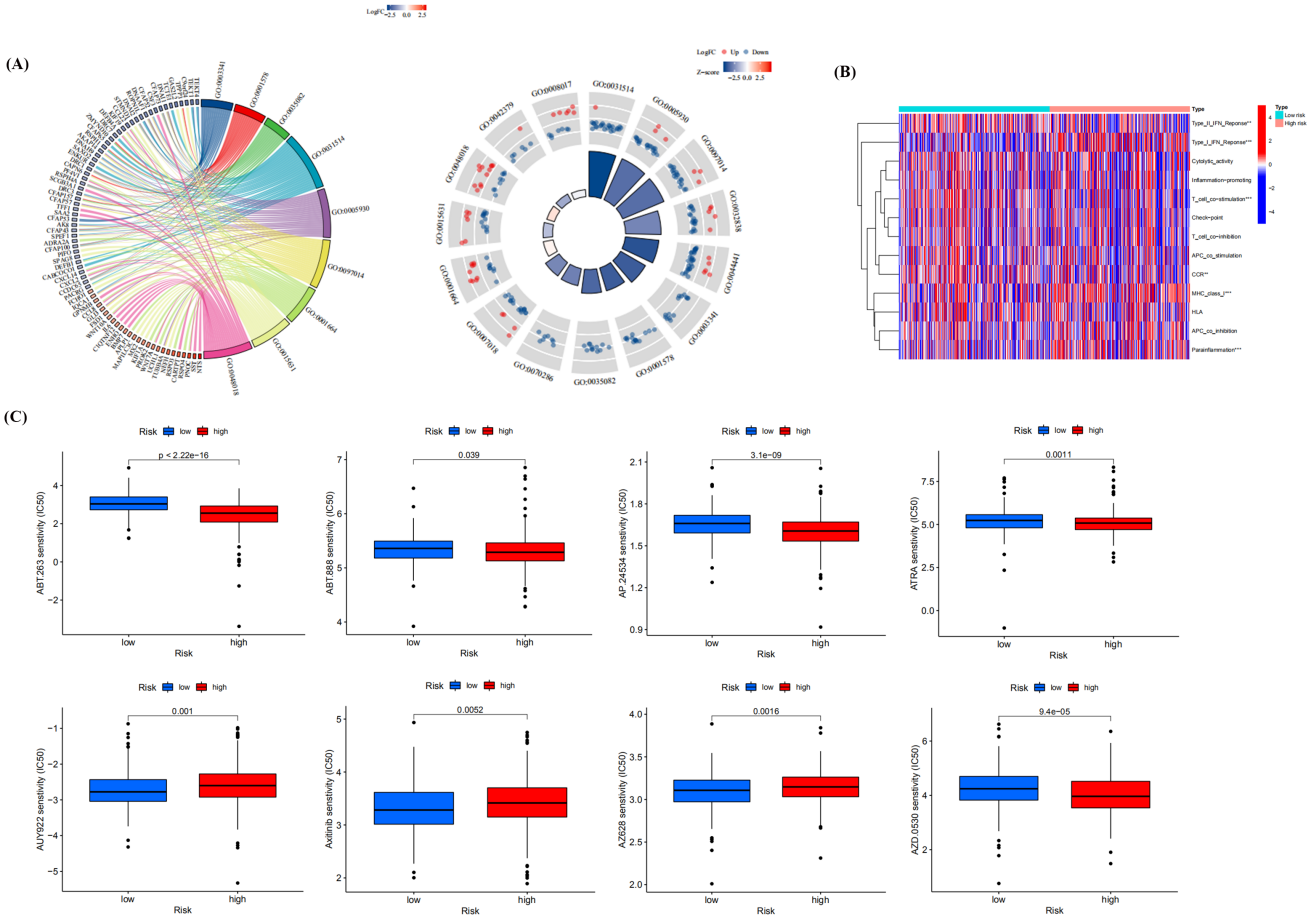
GO analysis of differential genes in the high‐ and low‐risk groups of the prognostic model, analysis of differential immune function, and analysis of drug sensitivity. (A) GO analysis of differential genes in the prognostic model's high‐ and low‐risk groups (circle and chordal plots). (B) Analysis of differences in immune function in the prognostic model (*p < 0.05; **p < 0.01; ***p < 0.001). (C) Drug sensitivity analysis of the prognostic model.

Figure 4A shows chordal and circle plots illustrating the GO analysis results for differentially expressed genes in the high‐ and low‐risk groups in the prognostic model. The first three items in the BP (biological process) analysis were cilium movement (GO:0003341), microtubule bundle formation (GO:0001578), and axoneme assembly (GO:0035082). The first three items in the CC (cellular component) analysis were motile cilium (GO:0031514), axoneme (GO:0005930), and ciliary plasm (GO:0097014). The first three items in the MF (molecular function) analysis were G protein‐coupled receptor binding (GO:0001664), tubulin binding (GO:0015631), and receptor–ligand activity (GO:0048018). The above GO analysis showed a *p* value of <0.05. Table S1 collates the GO analysis of genes differing between the high‐ and low‐risk groups in the prognostic model. We found that the immune function of parainflammation and the type I IFN response was more active in the high‐risk group than in the low‐risk group. In contrast, immune function was involved in T cell costimulation and the type II IFN response in the low‐risk group (Figure 4B). The prediction of potential therapeutic agents showed significant differences in sensitivity to eight chemicals between the low‐ and high‐risk groups (Figure 4C). Sensitivity to five compounds (ABT.263, ABT.888, AP.24534, ATRA, and AZD.0530) was found in the high‐risk group and to three compounds (AUY922, axitinib, and AZ628) was found in the low‐risk group. These findings may contribute to the clinical treatment of UCEC.

### 3.5 Survival curves for m6A‐associated lncRNAs, univariate and multivariate analysis, and nomogram construction

**FIGURE 5:**
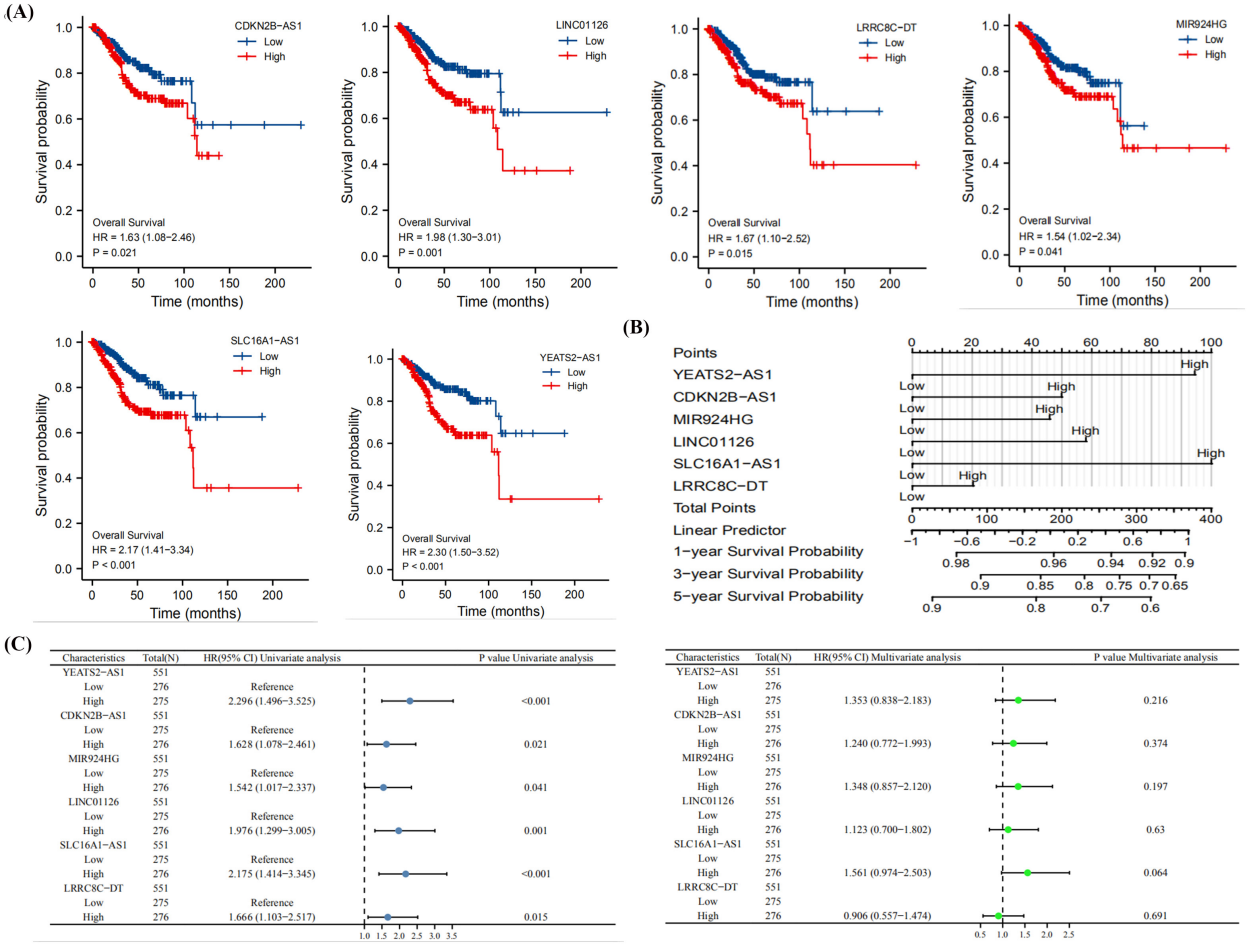
Survival curves and Nomogram construction of m6A‐associated lncRNAs. (A) Survival curves of m6A‐associated lncRNAs. (B) Nomogram plots of m6A‐associated lncRNAs. (C) Univariate and multivariate analysis of m6A‐associated lncRNAs

We used R software to plot the survival curves for prognosis‐associated m6A lncRNAs in EC to analyze the prognostic relationship between each m6A prognosis‐associated lncRNA and UCEC (Figure 5A). cdKN2B‐AS1 (HR = 1.63, 95% CI = 1.08–2.46, *p* = 0.021), YEATS2‐AS1 (HR = 2.30, 95% CI = 1.50–3.52, *p* < 0.001), MIR924HG (HR = 1.54, 95% CI = 1.02–2.34, *p* = 0.041), LRRC8C‐DT (HR = 1.67, 95% CI = 1.10–2.52, *p* = 0.015), SLC16A1‐AS1 (HR = 2.17, 95% CI = 1.41–3.34, *p* < 0.001), and LINC01126 (HR = 1.98, 95% CI = 1.30–3.01, *p* = 0.001) were all unfavorable factors for UCEC prognosis. Next, we plotted column line graphs containing risk classes and clinical risk characteristics to predict the incidence of OS at 1, 3, and 5 years (Figure 5B). Finally, we performed univariate and multivariate analyses of prognosis‐related m6A lncRNAs using R software (Figure 5C). In the univariate Cox regression analysis, YEATS2‐AS1, CDKN2B‐AS1, MIR924HG, SLC16A1‐AS1, LRRC8C‐DT, and LINC01126 were significantly differentially expressed in UCEC patients.

### 3.6 Clinical features of m6A‐related lncRNAs and EC and differences in the expression of m6A‐related lncRNAs in normal and tumor tissues

**FIGURE 6:**
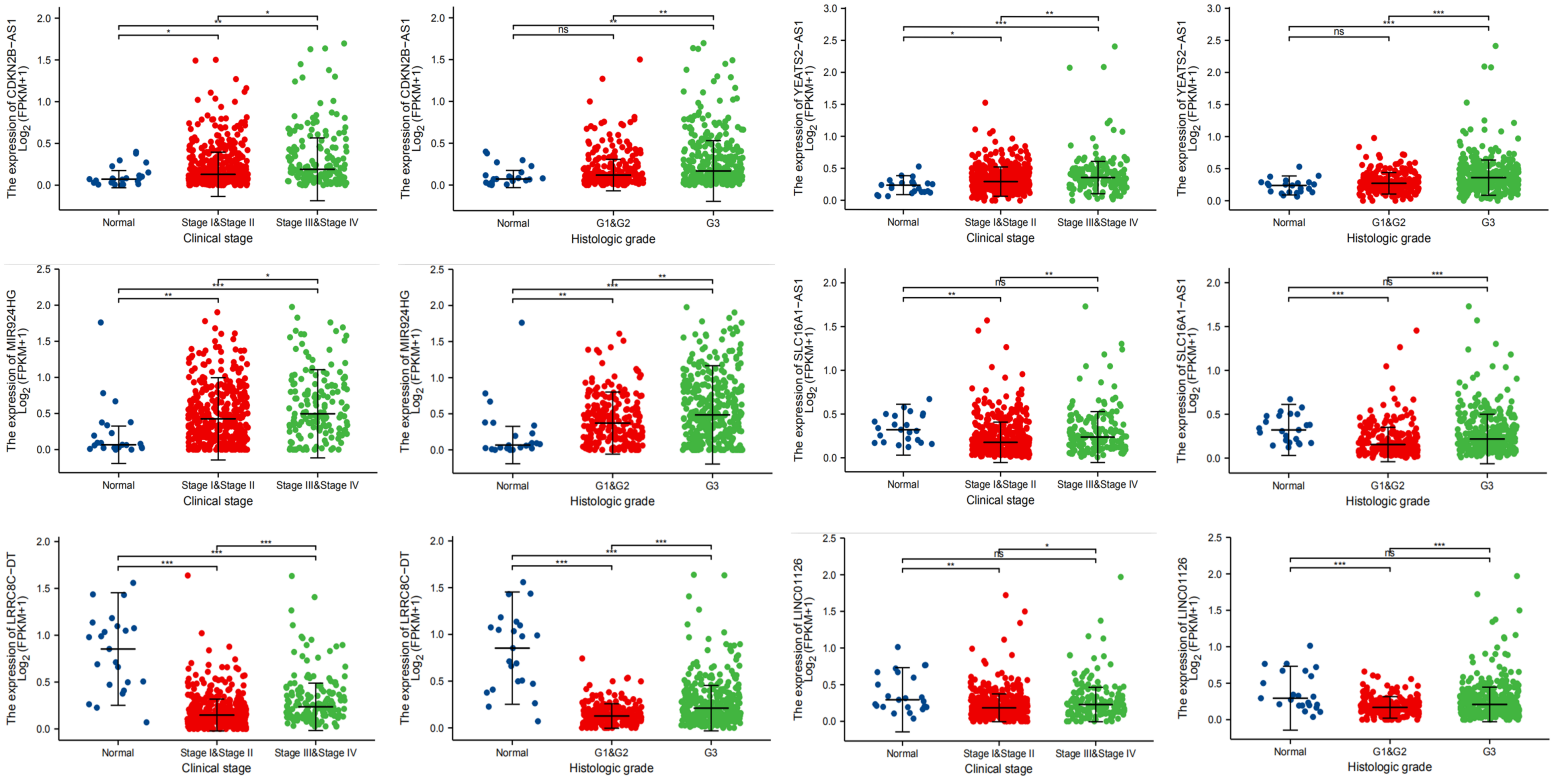
Differential expression of m6A‐related lncRNA in normal and EC tissues (by clinical stage and tumor grade) (ns, *p* ≥ 0.05; *, *p* < 0.05; **, *p* < 0.01; ***, *p* < 0.001).

**FIGURE 7:**
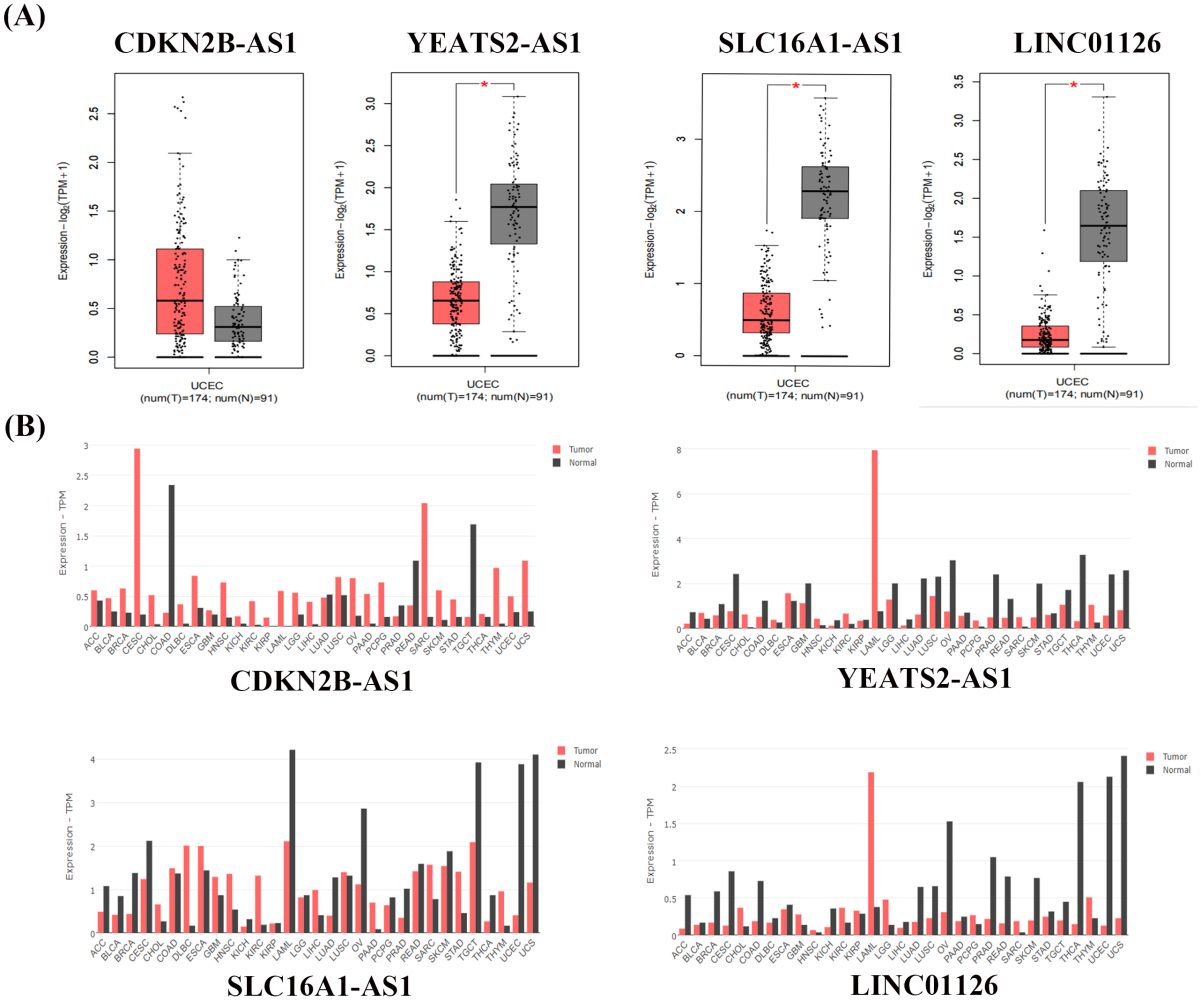
Differential expression of m6A‐related lncRNA in normal and tumor tissues. (A) Expression of m6A‐related LncRNA in EC and normal tissues in GEPIA database. (B) Expression of m6A‐related LncRNA in different tumors and normal tissues in GEPIA database.

We found that CDKN2B‐AS1, YEATS2‐AS1, MIR924HG, SLC16A1‐AS1, LRRC8C‐DT, and LINC01126 expression levels were significantly correlated with clinical stage and tumor grade in EC (Tables S2 and S3). CDKN2B‐AS1 and MIR924HG expression levels were higher in tumor tissues than in normal tissues and increased with clinical stage and tumor grade (Figure 6). SLC16A1‐AS1, LRRC8C‐DT, and LINC01126 expression was higher in normal tissues than in tumor tissues, and SLC16A1‐AS1, LRRC8C‐DT, and LINC01126 expression increased with an increase in clinical stage and tumor grade. The expression of CDKN2B‐AS1 was higher in cervical squamous cell carcinoma and endocervical adenocarcinoma (CESC) and in sarcoma (SARC) than in normal tissues. The expression of YEATS2‐AS1 and LINC01126 in acute myeloid leukemia (LAML) was higher than that in normal tissues (Figure 7B).

### 3.7 Immune infiltration of m6A‐associated lncRNAs in EC, intermolecular relationships, and mutations

**FIGURE 8:**
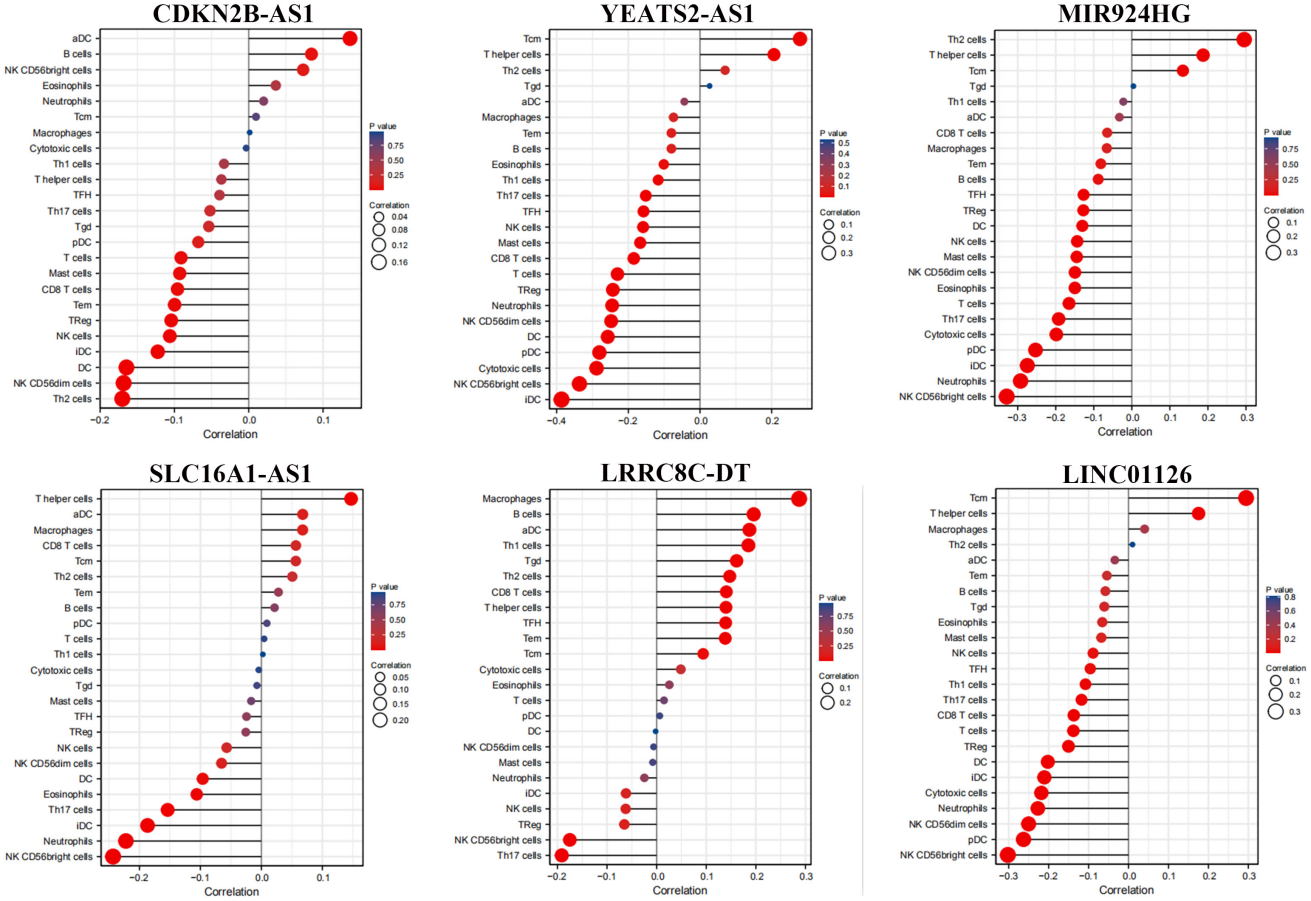
Immune cell infiltration of m6A‐related lncRNA in EC

**FIGURE 9:**
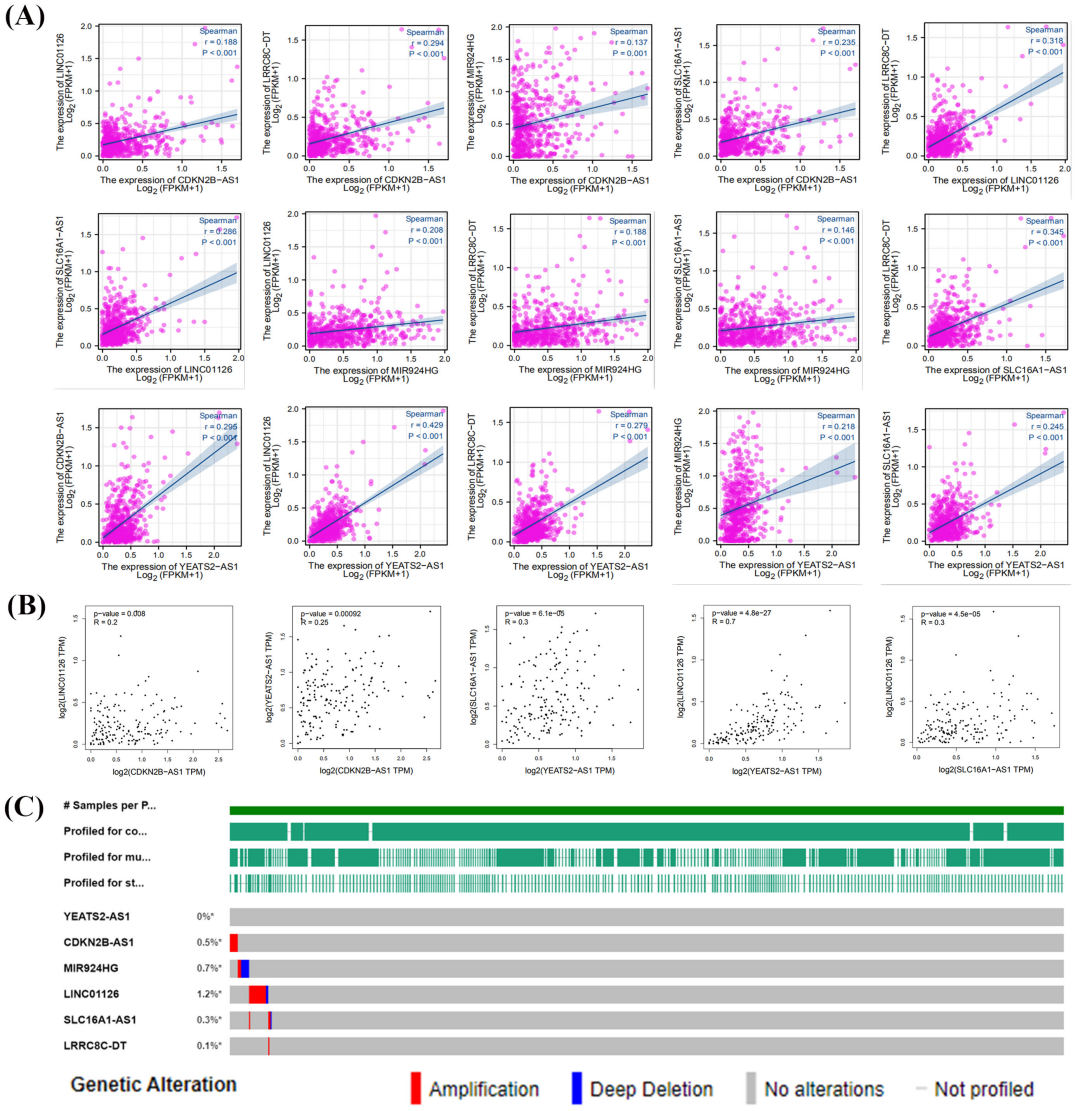
Intermolecular relationships and mutations of m6A‐associated lncRNAs. (A, B) Intermolecular relationships of m6A‐related lncRNAs in EC. (C) Mutation of m6A‐related lncRNA in EC

The relationship between m6A‐associated lncRNAs and immunological infiltration in EC was determined using the ssGSEA method and a Spearman correlation test (Figure 8). In UCEC, CDKN2B‐AS1 was negatively correlated with various types of immune cells, including DCs (*r* = −0.165), iDCs (*r* = −0.122), NK CD56dim cells (*r* = −0.168), and Th2 cells (*r* = −0.170). CDKN2B‐AS1 was positively correlated with aDCs (*r* = 0.136). LRRC8C‐DT was positively correlated with various types of immune cells, including aDCs (*r* = 0.187), B cells (*r* = 0.196), CD8 T cells (*r* = 0.140), and macrophages (*r* = 0.287). CD56bright cells (*r* = −0.175) and Th17 cells (*r* = −0.191) were negatively correlated with LRRC8C‐DT. We correlated the six prognosis‐related m6A lncRNAs with UCEC patient data from TCGA and GEPIA using Spearman's statistics (Figure 9A,B). We found that the six prognosis‐related m6A lncRNAs were positively correlated in EC (*p* < 0.05). YEATS2‐AS1 was positively correlated with LINC01126 (*r* = 0.429, *p* < 0.001), SLC16A1‐AS1 was positively correlated with LRRC8C‐DT (*r* = 0.345, *p* < 0.001), and CDKN2B‐AS1 was positively correlated with LRRC8C‐DT (*r* = 0.294, *p* < 0.001). We used the cBioPortal database to examine the mutation profile of the six m6A‐related lncRNAs in UCEC patients (Figure 9C). Except for YEATS2‐AS1, all other m6A‐related lncRNAs were mutated to varying degrees in UCEC (mutation rate < 2%). In 1445 UCEC patient samples, LINC01126 was mutated at a rate of 1.2% (17/1445), MIR924HG at a rate of 0.7% (10/1445), CDKN2B‐AS1 at a rate of 0.5% (7/1445), SLC16A1‐AS1 at a rate of 0.3% (4/1445), and LRRC8C‐DT at a rate of 0.1% (1/1445).

### 3.8 Analysis of the methylation level of m6A‐associated lncRNAs in EC, ceRNA network construction, and GO and KEGG analysis of lncRNA‐associated ceRNAs

**FIGURE 10:**
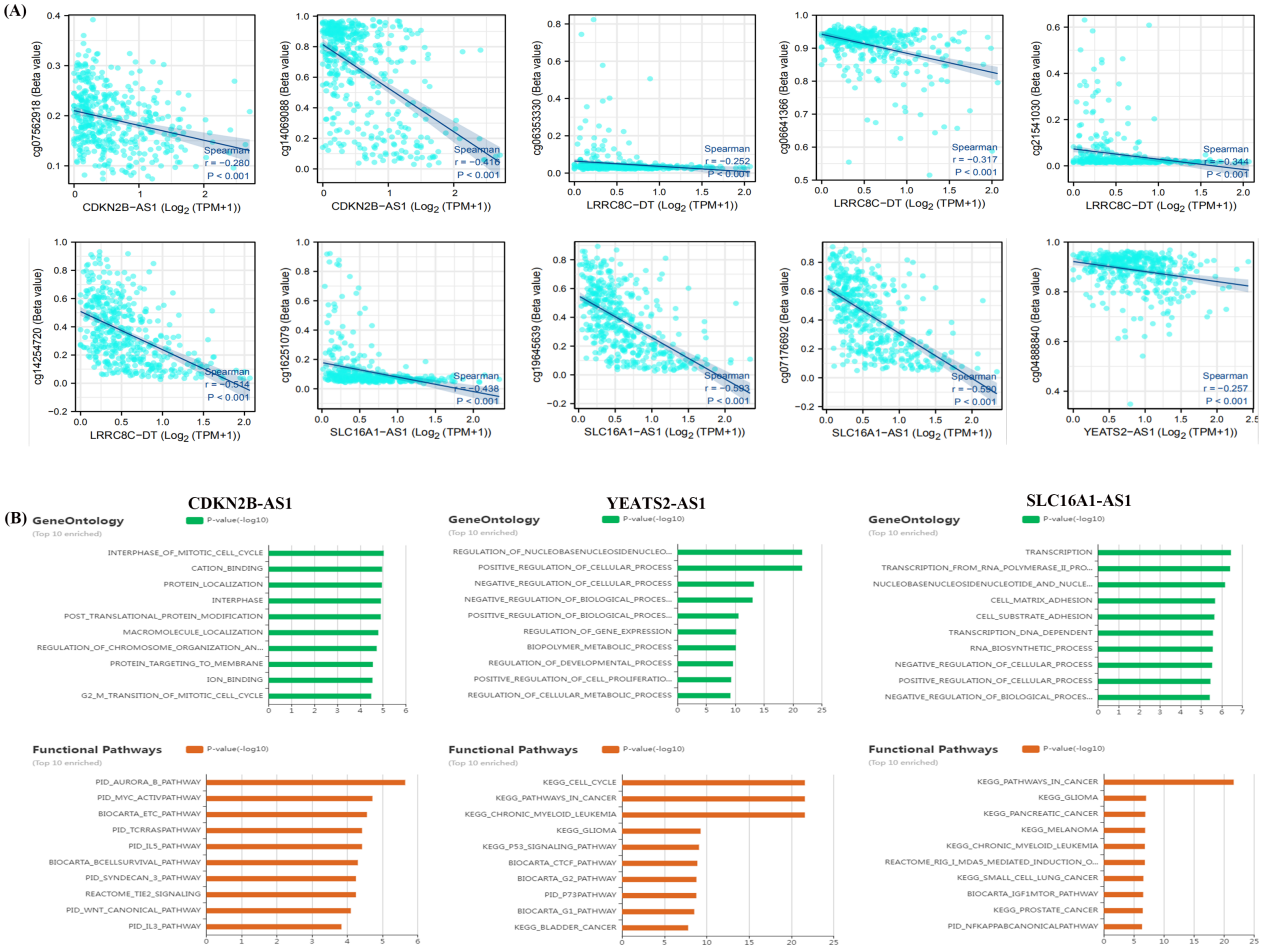
Methylation of m6A‐related lncRNA in EC and GO/KEGG analysis of m6A lncRNA‐associated ceRNAs. (A) Methylation of m6A‐related lncRNA. (B) GO and KEGG analysis of m6A lncRNA‐associated ceRNAs.

Methylation primarily occurs at CpG sites. Methylation is believed to occur in the promoter region and affects gene transcription. Beta values estimate the methylation degree and represent the signal intensity ratio between methylated and nonmethylated bases. Beta values range from 0 to 1, with 0 representing no methylation and 1 representing complete methylation. We found that m6A‐associated lncRNA expression in EC was negatively correlated with the corresponding methylation sites (Figure 10A). SLC16A1‐AS1 expression was negatively correlated with the methylation of cg07176692 (*r* = 0.590, *p* < 0.001) and cg19645639 (*r* = 0.593, *p* < 0.001) in EC. LRRC8C‐DT expression was negatively correlated with cg14254720 methylation (*r* = 0.514, *p* < 0.001) in EC. CDKN2B‐AS1 expression was negatively correlated with cg14069088 methylation (*r* = 0.416, *p* < 0.001) in EC.

We primarily performed GO and KEGG analyses of YEATS2‐AS1‐, CDKN2B‐AS1‐, and SLC16A1‐AS1‐related ceRNAs. We used the LncACTdb database for GO and KEGG analysis of m6A lncRNA‐associated ceRNAs to determine the dysregulated function of m6A lncRNA‐associated ceRNAs (Figure 10B). The GO analysis of YEATS2‐AS1‐associated ceRNAs focused on nucleobase, nucleoside, nucleotide, and nucleic acid metabolic process regulation. GO analysis of CDKN2B‐AS1‐related ceRNAs focused on mitotic cell cycle interphase. GO analysis of SLC16A1‐AS1‐associated ceRNAs focused on transcription.

**FIGURE 11:**
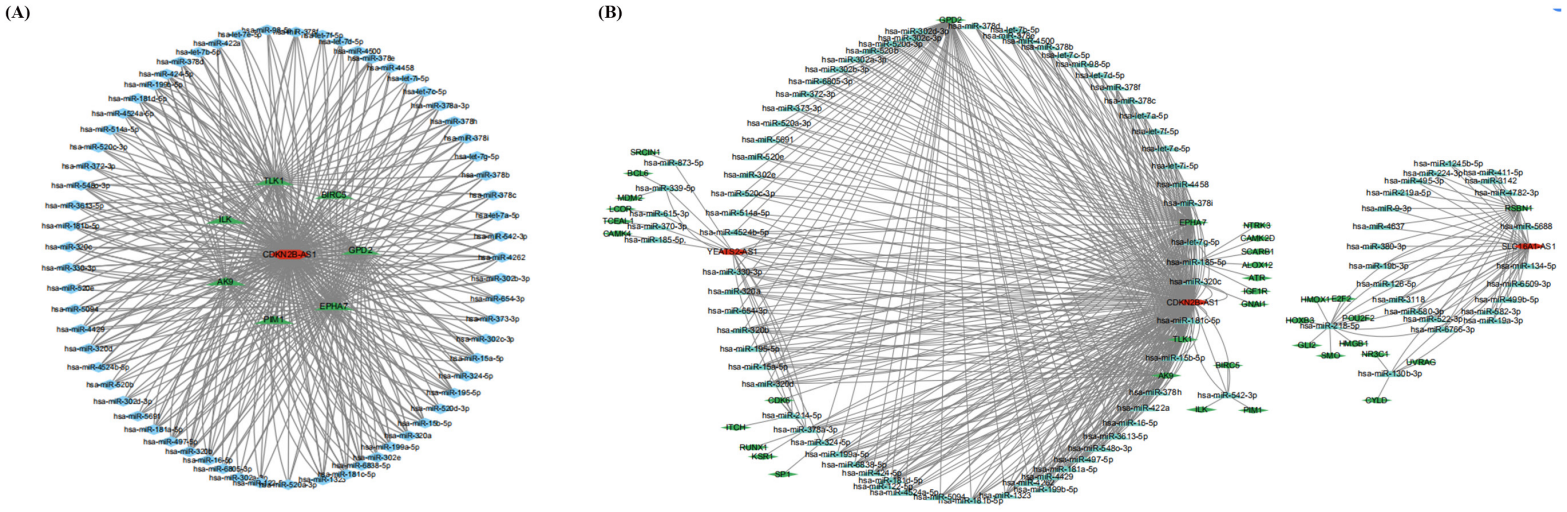
m6A lncRNA‐associated ceRNA constructs. (A) CDKN2B‐AS1‐associated ceRNA network construction. (B) Construction of CDKN2B‐AS1, YEATS2‐AS1, and SLC16A1‐AS1‐associated ceRNA networks

We used the Uterus project in the LncACTdb database to find ceRNAs associated with m6A lncRNAs, plotted the tables, and used Cytoscape software to construct the ceRNA network (Table S4). CDKN2B‐AS1 was found to have a competitive, regulatory relationship with mRNAs and miRNAs in endometrial cancer. For example, regulatory networks, such as CDKN2B‐AS1‐PIM1‐hsa‐miR‐542‐3p, CDKN2B‐AS1‐ILK‐hsa‐miR‐542‐3p, and CDKN2B‐AS1‐BIRC5‐hsa‐miR‐542‐3p, were identified (Figure 11A). pIM1, ILK, AS1, and CDKN2B‐AS1 had a competitive regulatory relationship with hsa‐miR‐542‐3p. Figure 11B depicts the ceRNA network construction associated with CDKN2B‐AS1, YEATS2‐AS1, and SLC16A1‐AS1. The ceRNA networks of CDKN2B‐AS1 and YEATS2‐AS1 intersect, while the ceRNA network of SLC16A1‐AS1 is relatively independent.

### 3.9 CCLE database bioinformatics analysis and qRT‐PCR experimental validation of m6A‐related lncRNAs

**FIGURE 12:**
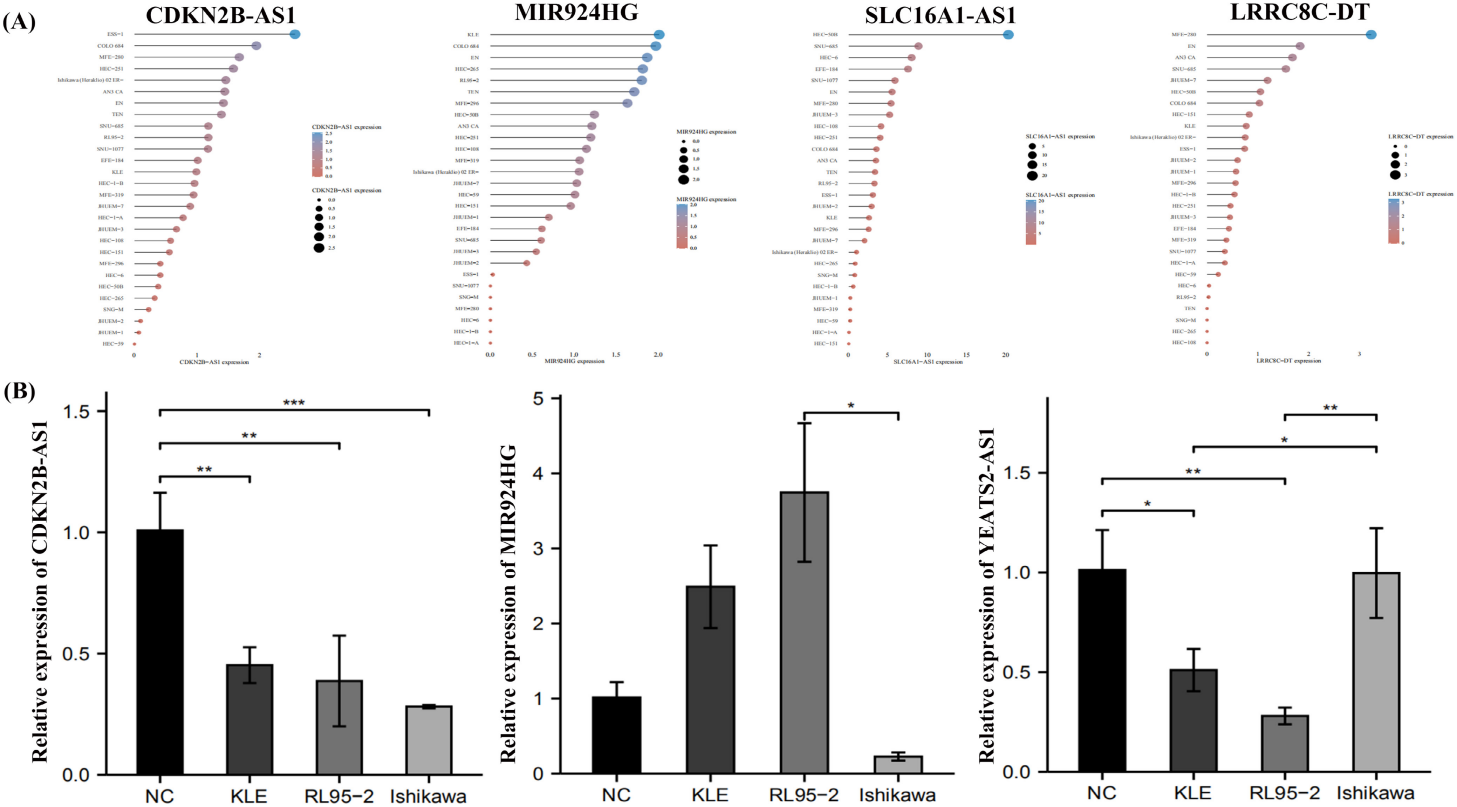
Expression of m6A‐associated lncRNA in different endometrial cancer cell lines. (A) Expression of m6A‐related lncRNA in different endometrial cancer cell lines from CCLE database. (B) Validation of m6A‐related lncRNA by qRT‐PCR cell assay

We explored m6A‐related lncRNA expression in different endometrial cancer cell lines using bioinformatics analysis and the CCLE database (Figure 12A). We found that CDKN2B‐AS1 was highly expressed in ESS‐1 and COLO 684 cells. MIR924HG was highly expressed in KLE and COLO 684 cells. LRRC8C‐DT and SLC16A1‐AS1 were highly expressed in MFE‐280 and HEC‐50B cells, respectively. We used endometrial epithelial cells and endometrial cancer cells (RL95‐2, Ishikawa, and KLE cells) to validate the bioinformatics findings. Table S5 shows the m6A‐related lncRNA primer sequences. Quantitative real‐time polymerase chain reaction (qRT‐PCR) results showed that CDKN2B‐AS1 and YEATS2‐AS1 expression was downregulated in KLE and RL95‐2 cells, whereas MIR924HG expression was upregulated in KLE and RL95‐2 cells (Figure 12B).

## 4 DISCUSSION

Endometrial carcinoma is one of the three most prevalent malignancies in females and is characterized by angiogenesis, chronic inflammation, and a high degree of immunogenicity. EC exhibits variable sensitivity to antiangiogenic drugs and immunotherapy. Some studies have suggested that biomarkers and predictive models can improve targeted therapy and immunotherapy in oncology patients.^4^ However, the exploration of risk prediction models remains a challenge for precision medicine options for UCEC patients. Recent studies have shown that m6A‐related lncRNAs are closely associated with pathophysiological processes in various tumors, including pancreatic cancer, colon cancer, HCC, acute myeloid leukemia, and bladder cancer. lncRNA expression affects prognosis in multiple cancer types and is expected to be a tumor biomarker. However, it is unclear whether m6A‐related lncRNAs are associated with UCEC prognosis. This study aimed to construct a prognostic model of m6A‐related lncRNAs, perform bioinformatics analyses, and experimentally validate m6A‐related lncRNAs in UCEC.

We identified m6A‐associated lncRNAs in 552 EC and 23 normal tissue samples using Perl and R software and mapped m6A‐associated mRNAs and lncRNA coexpression in a Sankey map. We screened 32 m6A lncRNAs associated with EC prognosis. The LASSO method was used to construct multivariate proportional risk regression models (RAB11B‐AS1, LINC01812, HM13‐IT1, SLC16A1‐AS1, TPM1‐AS, LINC01936, and CDKN2B‐AS1). We divided the UCEC patients into high‐ and low‐risk groups and plotted ROC curves and performed PCA of the constructed prognostic models. The AUC for our constructed prognostic model was predictive, and the model effectively distinguished between low‐ and high‐risk groups. Next, we performed GO analysis of differentially expressed genes between the high‐ and low‐risk groups, immune function differential analysis, and drug sensitivity analysis of the prognostic model. The relationship between the prognostic model and tumor immunity was explored to predict potential drugs that could be used to treat UCEC.

In addition to constructing a prognostic model for EC, we performed an in‐depth analysis of the association between individual prognosis‐related m6A lncRNAs and EC, survival curve mapping, univariate and multivariate analyses, and nomogram plot construction. Bioinformatics analysis revealed that CDKN2B‐AS1, YEATS2‐AS1, MIR924HG, SLC16A1‐AS1, LRRC8C‐DT, and LINC01126 expression was significantly associated with clinical stage and tumor grade in EC. CDKN2B‐AS1 and MIR924HG were expressed at higher levels in endometrial cancer tissues than in normal tissues. Finally, we performed immunoinfiltration, methylation, analysis of m6A‐related lncRNAs in EC, and ceRNA network construction to further investigate the m6A‐related lncRNA mechanism in endometrial carcinogenesis and development. Identifying mRNA‐lncRNA‐miRNA regulatory relationships in EC can facilitate the study of the molecular mechanisms underlying EC development, optimize diagnosis and treatment of the disease, and aid in development of targeted drugs.

Bioinformatics analysis revealed that YEATS2‐AS1, CDKN2B‐AS1, and MIR924HG are risk factors for EC, while RAB11B‐AS1 is a protective factor for EC patients among the m6A lncRNAs associated with EC prognosis. YEATS2‐AS1 is a risk factor in various tumors. YEATS2‐AS1 is differentially expressed in prostate cancer and is a high‐risk factor for prostate cancer.^25^ Zhang et al. suggested that YEATS2‐AS1 can be used as a biomarker to predict OS in sarcoma patients, providing new insights into the treatment of soft tissue sarcoma (STS). YEATS2‐AS1 expression was found to be significantly higher in STS tissues than in normal tissues, and higher YEATS2‐AS expression was associated with lower OS in STS patients.^26^ The GEPIA database showed that the expression of YEATS2‐AS1 was higher in normal tissues, but the survival curve showed that the prognosis of EC patients with high YEATS2‐AS1 expression was worse. If YEATS2‐AS1 is an oncogene, the smaller the degree of inhibition in the cancer tissue is, the higher the expression, and the worse the prognosis of the patient. If YEATS2‐AS1 is a tumor suppressor gene, the higher the malignancy of cancer cells is, the more tumor suppressor genes are needed, and the higher the expression. The molecular mechanisms of YEATS2‐AS1 and MIR924HG in EC have not yet been explored and may provide directions for future studies.

CDKN2B‐AS1 is aberrantly expressed in various malignancies, including HCC, cervical cancer, ovarian cancer, and breast cancer. It is involved in processes such as tumor cell proliferation, migration, invasion, and apoptosis inhibition.^27^ Yang et al.^28^ reported that CDKN2B‐AS1 is overexpressed in EC. CDKN2B‐AS1 knockdown inhibited the proliferation and invasion of EC cells (Ishikawa and HEC‐1A cells) and suppressed transplanted tumor growth in nude mice, suggesting that CDKN2B‐AS1 is a risk factor for EC, consistent with our study. CDKN2B‐AS1 also plays an oncogenic role in other gynecological tumors, such as cervical and ovarian cancers. Researchers have demonstrated the role of CDKN2B‐AS in cervical cancer through in vivo experiments, including tumor growth, apoptosis inhibition, and senescence inhibition, and CDKN2B‐AS1 knockdown can inhibit the above activities.^29^ In vitro and in vivo, CDKN2B‐AS1 interacts with miR‐411‐3p to regulate ovarian cancer through the HIF‐1a/VEGF/P38 pathway. sh‐CDKN2B‐AS1 showed anticancer effects by promoting apoptosis and inhibiting cell growth, invasion, and migration.^30^ In addition to malignancies, CDKN2B‐AS1 is associated with various nonmalignant diseases, including idiopathic pulmonary fibrosis, endometriosis, diabetes, and coronary artery disease.^27^ Relative CDKN2B‐AS1 expression was upregulated in situ and in ectopic endometrium. miR‐424‐5p attenuated the CDKN2B‐AS1 effect on cell proliferation, invasion, and AKT3 expression in an ovarian endometriosis model.^31^ CDKN2B‐AS1 may be a potential target for ovarian endometriosis therapy. In summary, we suggest that CDKN2B‐AS1 may serve as a therapeutic target or prognostic biomarker of various human diseases.

RAB11B‐AS1 may play different roles in different tumors. Previous studies have pointed to a higher survival rate of EC patients with high RAB11B‐AS1 expression, and RAB11B‐AS1 appears to be a prognostic factor in EC. The lncRNA RAB11B‐AS1 is negatively correlated with the EC malignant phenotype.^32^, ^33^ This is consistent with our findings. RAB11B‐AS1 increased breast cancer cell migration and invasion in vitro and promoted tumor angiogenesis and distant breast cancer metastasis without affecting primary tumor growth in mice. Hypoxia‐inducible factor 2 (HIF2)‐induced RAB11B‐AS1 promoted hypoxia‐mediated angiogenesis and breast cancer metastasis.^34^ In HCC, RAB11B‐AS1 is a protective factor. RAB11B‐AS1 inhibited HCC cell proliferation, migration, and invasion in vivo; promoted HCC cell apoptosis; and inhibited HCC tumor growth. METTL16 promoted HCC progression by downregulating RAB11B‐AS1 expression in an m6A‐dependent manner. The METTL16/RAB11B‐AS1 regulatory axis in HCC is expected to be a new target in HCC prognosis assessment and treatment.^35^

Regarding immune function, we found that parainflammation and type I IFN responses were more active in the high‐risk group than in the low‐risk group in the prognostic model. Chronic inflammation has been recognized as one of the hallmarks of cancer. Aran et al. showed that parainflammation, a distinct inflammatory variant between homeostasis and chronic inflammation, is widely prevalent in human cancer and can strongly promote intestinal tumorigenesis in mice following p53 deletion.^36^, ^37^ This study points to parainflammation as a possible driver of p53 mutagenesis and promises cancer prevention through NSAID treatment. Our study showed that parainflammation was more active in the high‐risk group for EC, suggesting its involvement in cancer progression. We consider type I interferons (IFN‐Is) essential drivers of antitumor immunity, effectively stimulating immune cells and thus eliminating tumor cells. However, it has been suggested that IFN‐Is have a more complex role and that prolonged stimulation can promote inhibitory feedback mechanisms that lead to immune failure, directly or indirectly allowing cancer cells to evade immune clearance, with deleterious effects on the body.^38^ Specifically, the type I IFN response induces numerous immune dysfunctions throughout the immune response in times of chronic disease, thereby impeding cancer control. There are indications that IFN‐Is can play a negative role by promoting negative feedback and immunosuppression. Sustained IFN‐I signaling may be a key driver of immune dysfunction in some cancer types.^39^ In the present study, the type I IFN response was more active in the high‐risk group for EC, suggesting that it may exert negative feedback and play an immunosuppressive role in EC development.

We performed drug sensitivity analysis of the prognostic models constructed to predict potential drugs that could be used to treat UCEC. The high‐risk group was more sensitive to five compounds (ABT.263, ABT.888, AP24534, ATRA, and AZD.0530) than the low‐risk group. It has been suggested that the poly (ADP‐ribose) polymerase (PARP) inhibitor veriparib (ABT‐888) has potential clinical application as a radiosensitizer in the pharmacological treatment of EC. Veriparib combined with radiotherapy significantly slowed tumor growth and improved treatment efficiency in EC compared to monotherapy with either veriparib or radiotherapy.^40^ PARP1 inhibitors (ABT‐888) are also of selective value in other gynecological tumors. Innovative treatment of epithelial ovarian cancer with PARP inhibitors (PARPis) has shown excellent effectiveness, plays a vital role in the treatment of newly diagnosed ovarian cancer, and is changing clinical practice in patients with BRCA mutations.^41^, ^42^ Kim et al. noted aberrant mutational FGFR2 activation associated with EC.

AP24534 (ponatinib), an orally available multitarget tyrosine kinase inhibitor currently in clinical trials, exerts its chemotherapeutic effects primarily by blocking ERK, PLCγ, and STAT5 signaling in EC.^43^ This study showed that AP24534 inhibited the migration and invasion of FGFR2‐mutated endometrial cancer cells and had antiendometrial cancer effects. All‐trans retinoic acid (ATRA) affects tumor progression in the human endometrial cancer cell lines RL95‐2 and Hec1A. Tsuji et al. found that ATRA inhibited cell proliferation, apoptosis, and migration in RL95‐2 cells but not in Hec1A cells. Tsuji et al.^44^ suggested that retinoic acid (RA) may have multiple antitumor effects and that RARβ, which is regulated by ATRA, may be a potential RA target for EC treatment. These findings are consistent with our findings that high‐risk EC patients are more sensitive to ABT.888, AP24534, and ATRA.

The therapeutic role of ABT‐263 has been reported in other tumors, but no study has yet explored its therapeutic role in EC. ABT‐263 is a potent BH3 mimetic with anticancer potential in various cancer types. This potential is due to its high binding affinity for anti‐apoptotic proteins in the Bcl‐2 family. It has been noted that ABT‐263 exhibits apoptosis‐inducing potential in oral cancer cells by targeting C/EBP‐homologous proteins.^45^ Tse et al. reported that ABT‐263 promotes apoptosis by inducing Bax translocation and cytochrome c release in human tumor cells. ABT‐263 oral administration caused complete tumor regression in xenograft models of small cell lung cancer and acute lymphoblastic leukemia.^46^

The forest plot shows 32 prognosis‐related m6A lncRNAs in EC. We selected six m6A‐associated lncRNAs for survival curve mapping and univariate and multivariate analyses. In the univariate analysis, we found significant expression differences in YEATS2‐AS1, CDKN2B‐AS1, MIR924HG, SLC16A1‐AS1, LRRC8C‐DT, and LINC01126 in UCEC patients, suggesting that these six m6A‐associated lncRNAs may be risk factors for EC. Bioinformatics analysis indicated that CDKN2B‐AS1 and MIR924HG expression is significantly higher in EC than in normal tissues, and the expression increased with clinical stage and tumor grade. We used qRT‐PCR to verify the expression of three m6A‐related lncRNAs in normal cells and EC cells. The qRT‐PCR results showed that CDKN2B‐AS1 and YEATS2‐AS1 expression was downregulated in KLE and RL95‐2 cells, while MIR924HG expression was upregulated in KLE and RL95‐2 cells. The low expression of CDKN2B‐AS1 in tumor cell lines seems to contradict its high expression in tumor tissues. Here, we attempted to analyze the possible causes. First, tissues are composed of many different cells, and the expression of lncRNAs differs in different cell lines. The expression in tissue reflects the general trend of the expression in all cells. The cell line we selected may be a cell line with low lncRNA expression. Second, Peng et al.^47^ explored the similarity between different cancer research models and primary tumors, thereby improving the cancer research model used in the laboratory. The research results showed that, among several commonly used tumor research models, human cancer cell lines grown in culture dishes are the most genetically dissimilar to human cancer cells and genetically engineered mice and 3D tumor models have more similarities with human cancer cells. Because of the complex differences between the growth environments, cancer cell lines grown in the laboratory are different from those in humans. Therefore, the cancer cell line model we studied may have changed compared with cancer cells in human tissues. Finally, the small sample size and confounding factors may have affected the experimental results.

SLC16A1‐AS1, LRRC8C‐DT, and LINC01126 expression was higher in normal tissues than in EC tissues, which appears to contradict our previous conclusion that these m6A‐related lncRNAs may be risk factors for EC. However, SLC16A1‐AS1, LRRC8C‐DT, and LINC01126 expression increased with clinical stage and endometrial cancer grade. This suggests that these m6A‐related lncRNAs may be involved in EC progression, and the exact mechanism of action needs to be further investigated using in vitro and in vivo experiments. A new gene regulatory program was revealed by Logotheti et al.,^48^ in which E2F1‐inducible SLC16A1‐AS1 forms a complex with its transcription factors to promote cancer metabolic reprogramming to obtain a mixed oxidative profile that favors bladder cancer (BC) aggressiveness. SLC16A1‐AS1 acts as a target and coactivator of E2F1 to induce metabolic reprogramming during BC progression. In vitro experiments conducted by Zhou et al.^49^ showed that LINC01126 inhibits proliferation and promotes apoptosis and inflammation in human periodontal cells under hypoxic conditions through miR‐518a‐5p. LINC01126 promotes the pathogenesis of periodontitis in human periodontal membrane cells via the miR‐518a‐5p/HIF‐1α/MAPK pathway, providing possible clues for LINC01126‐based therapeutic approaches for periodontal disease.

Our study has limitations and weaknesses. First, we only verified the differential expression of m6A‐related lncRNAs via qRT‐PCR in cultured cells and did not investigate functional mechanisms. Follow‐up in vivo and in vitro experiments are needed to further investigate the molecular mechanisms of m6A‐related lncRNA biological functions. Second, although lncRNA expression levels were measured using qRT‐PCR in three endometrial cancer cell lines and NEECs, the sample size was small, and more samples would improve the reliability of the findings. Third, we only validated our findings through TCGA database, and due to the lack of lncRNA expression profiles and OS data in other databases, other databases were not examined for joint validation. Finally, in retrospective studies, there may be some bias in case inclusion and data processing, and the effect of various confounding factors cannot be entirely excluded.

## 5 CONCLUSIONS

We constructed and validated a prognostic model consisting of seven m6A‐associated lncRNAs through bioinformatics analysis. Our prognostic model predicted OS and response to drug therapy in UCEC. Obtaining EC markers associated with prognosis and drug therapy response can improve targeted therapy and immunotherapy for EC patients. The bioinformatics analysis results suggested that CDKN2B‐AS1 and MIR924HG are risk factors for EC and that RAB11B‐AS1 is a protective factor in EC patients. m6A lncRNA expression was influenced by the type of endometrial cancer cell line. Identifying the mRNA‐lncRNA‐miRNA regulatory relationship in EC is beneficial for exploring the molecular mechanisms underlying EC development and progression and for developing targeted drugs.

## AUTHOR CONTRIBUTIONS

Writing—original manuscript preparation—was done by ZX; conceptualizing was done by ZX and ZH; material curation and deep analysis were done by ZX, ZH; and writing—review and editing—was done by DH, DY, and ZJ. All contributors have reviewed and approved the completed version of the article.

## FUNDING INFORMATION

The Discipline Construction Promoting Project of Shanghai Pudong Hospital (project no. Zdzk2020‐16 and Zdzk2020‐18). Key Specialty Construction Project of Pudong Health and Family Planning Commission of Shanghai, Grant/Award Number: PWZzk2022‐21.

## CONFLICT OF INTEREST

The authors have no conflicts of interest to declare.

## Supporting information


Table S1.

Table S2.

Table S3.

Table S4.

Table S5.

## Data Availability

The original contributions presented in the study are included in the article/Supplementary Material. Further inquiries can be directed to the corresponding authors.
