# Supplementary material for: m6A‐related long noncoding RNAs predict prognosis and indicate therapeutic response in endometrial carcinoma
Source: J Clin Lab Anal. 2022 Dec 16;37(1):e24813. doi: 10.1002/jcla.24813 (PMC9833960; doi:10.1002/jcla.24813)
Supplement: Supplementary file 1 — Table S1. [file JCLA-37-e24813-s003.docx]

**Table S1 GO analysis of differential genes in high and low risk groups in prognostic models**

| **ONTOLOGY** | **ID** | **Description** | ***p*value** | **Count** |
| --- | --- | --- | --- | --- |
| BP | GO:0003341 | cilium movement | 7.95E-12 | 14 |
| BP | GO:0001578 | microtubule bundle formation | 4.83E-10 | 14 |
| BP | GO:0035082 | axoneme assembly | 5.44E-10 | 12 |
| BP | GO:0070286 | axonemal dynein complex assembly | 5.14E-08 | 8 |
| BP | GO:0007018 | microtubule-based movement | 1.07E-07 | 20 |
| CC | GO:0031514 | motile cilium | 1.51E-15 | 25 |
| CC | GO:0005930 | axoneme | 4.76E-15 | 21 |
| CC | GO:0097014 | ciliary plasm | 5.64E-15 | 21 |
| CC | GO:0032838 | plasma membrane bounded cell projection cytoplasm | 3.21E-12 | 23 |
| CC | GO:0044441 | ciliary part | 1.33E-11 | 33 |
| MF | GO:0001664 | G protein-coupled receptor binding | 1.00E-05 | 17 |
| MF | GO:0015631 | tubulin binding | 2.98E-05 | 18 |
| MF | GO:0048018 | receptor ligand activity | 1.32E-04 | 21 |
| MF | GO:0042379 | chemokine receptor binding | 1.55E-04 | 7 |
| MF | GO:0008017 | microtubule binding | 4.31E-04 | 13 |
