# Supplementary material for: m6A‐related long noncoding RNAs predict prognosis and indicate therapeutic response in endometrial carcinoma
Source: J Clin Lab Anal. 2022 Dec 16;37(1):e24813. doi: 10.1002/jcla.24813 (PMC9833960; doi:10.1002/jcla.24813)
Supplement: Supplementary file 2 — Table S2. [file JCLA-37-e24813-s004.docx]

**Table S2 Relationship between CDKN2B-AS1, YEATS2-AS1, MIR924HG expression and clinical features of UCEC**

| **Characteristic** | Low expression of CDKN2B-AS1 | High expression of CDKN2B-AS1 | ***p*** | Low expression of YEATS2-AS1 | High expression of YEATS2-AS1 | ***p*** | Low expression of MIR924HG | High expression of MIR924HG | ***p*** |
| --- | --- | --- | --- | --- | --- | --- | --- | --- | --- |
| n | 276 | 276 |  | 276 | 276 |  | 276 | 276 |  |
| Clinical stage, n (%) |  |  | **0.045** |  |  | **0.020** |  |  | **0.018** |
| I | 186 (33.7%) | 156 (28.3%) |  | 184 (33.3%) | 158 (28.6%) |  | 187 (33.9%) | 155 (28.1%) |  |
| II | 25 (4.5%) | 26 (4.7%) |  | 29 (5.3%) | 22 (4%) |  | 21 (3.8%) | 30 (5.4%) |  |
| III | 54 (9.8%) | 76 (13.8%) |  | 52 (9.4%) | 78 (14.1%) |  | 52 (9.4%) | 78 (14.1%) |  |
| IV | 11 (2%) | 18 (3.3%) |  | 11 (2%) | 18 (3.3%) |  | 16 (2.9%) | 13 (2.4%) |  |
| Age, n (%) |  |  | **< 0.001** |  |  | **0.002** |  |  | 1.000 |
| <=60 | 132 (24%) | 74 (13.5%) |  | 121 (22%) | 85 (15.5%) |  | 103 (18.8%) | 103 (18.8%) |  |
| >60 | 141 (25.7%) | 202 (36.8%) |  | 153 (27.9%) | 190 (34.6%) |  | 172 (31.3%) | 171 (31.1%) |  |
| Histological type, n (%) |  |  | **< 0.001** |  |  | **< 0.001** |  |  | **0.014** |
| Endometrioid | 249 (45.1%) | 161 (29.2%) |  | 235 (42.6%) | 175 (31.7%) |  | 220 (39.9%) | 190 (34.4%) |  |
| Mixed | 5 (0.9%) | 19 (3.4%) |  | 9 (1.6%) | 15 (2.7%) |  | 10 (1.8%) | 14 (2.5%) |  |
| Serous | 22 (4%) | 96 (17.4%) |  | 32 (5.8%) | 86 (15.6%) |  | 46 (8.3%) | 72 (13%) |  |
| Histologic grade, n (%) |  |  | **< 0.001** |  |  | **< 0.001** |  |  | **0.005** |
| G1 | 47 (8.7%) | 51 (9.4%) |  | 65 (12%) | 33 (6.1%) |  | 62 (11.5%) | 36 (6.7%) |  |
| G2 | 81 (15%) | 39 (7.2%) |  | 75 (13.9%) | 45 (8.3%) |  | 63 (11.6%) | 57 (10.5%) |  |
| G3 | 145 (26.8%) | 178 (32.9%) |  | 134 (24.8%) | 189 (34.9%) |  | 145 (26.8%) | 178 (32.9%) |  |
| Overall survival （OS）, n (%) |  |  | 0.054 |  |  | **0.001** |  |  | **0.031** |
| Survive | 238 (43.1%) | 220 (39.9%) |  | 244 (44.2%) | 214 (38.8%) |  | 239 (43.3%) | 219 (39.7%) |  |
| Death | 38 (6.9%) | 56 (10.1%) |  | 32 (5.8%) | 62 (11.2%) |  | 37 (6.7%) | 57 (10.3%) |  |
