# Supplementary material for: m6A‐related long noncoding RNAs predict prognosis and indicate therapeutic response in endometrial carcinoma
Source: J Clin Lab Anal. 2022 Dec 16;37(1):e24813. doi: 10.1002/jcla.24813 (PMC9833960; doi:10.1002/jcla.24813)
Supplement: Supplementary file 3 — Table S3. [file JCLA-37-e24813-s001.docx]

**Table S3 Relationship between SLC16A1-AS1, LRRC8C-DT, LINC01126 expression and clinical features of UCEC**

| **Characteristic** | Low expression of **SLC16A1-AS1** | High expression of SLC16A1-AS1 | ***p*** | Low expression of LRRC8C-DT | High expression of LRRC8C-DT | | ***p*** | Low expression of **LINC01126** | High expression of LINC01126 | ***p*** |
| --- | --- | --- | --- | --- | --- | --- | --- | --- | --- | --- |
| n | 276 | 276 |  | 276 | 276 |  | | 276 | 276 |  |
| Clinical stage, n (%) |  |  | **0.001** |  |  | **< 0.001** | |  |  | **0.048** |
| I | 192 (34.8%) | 150 (27.2%) |  | 196 (35.5%) | 146 (26.4%) |  | | 186 (33.7%) | 156 (28.3%) |  |
| II | 22 (4%) | 29 (5.3%) |  | 26 (4.7%) | 25 (4.5%) |  | | 23 (4.2%) | 28 (5.1%) |  |
| III | 54 (9.8%) | 76 (13.8%) |  | 46 (8.3%) | 84 (15.2%) |  | | 57 (10.3%) | 73 (13.2%) |  |
| IV | 8 (1.4%) | 21 (3.8%) |  | 8 (1.4%) | 21 (3.8%) |  | | 10 (1.8%) | 19 (3.4%) |  |
| Age, n (%) |  |  | **0.006** |  |  | **0.019** | |  |  | 0.143 |
| <=60 | 119 (21.7%) | 87 (15.8%) |  | 117 (21.3%) | 89 (16.2%) |  | | 112 (20.4%) | 94 (17.1%) |  |
| >60 | 155 (28.2%) | 188 (34.2%) |  | 158 (28.8%) | 185 (33.7%) |  | | 163 (29.7%) | 180 (32.8%) |  |
| Histological type, n (%) |  |  | **< 0.001** |  |  | **< 0.001** | |  |  | **0.001** |
| Endometrioid | 230 (41.7%) | 180 (32.6%) |  | 246 (44.6%) | 164 (29.7%) |  | | 224 (40.6%) | 186 (33.7%) |  |
| Mixed | 10 (1.8%) | 14 (2.5%) |  | 8 (1.4%) | 16 (2.9%) |  | | 9 (1.6%) | 15 (2.7%) |  |
| Serous | 36 (6.5%) | 82 (14.9%) |  | 22 (4%) | 96 (17.4%) |  | | 43 (7.8%) | 75 (13.6%) |  |
| Histologic grade, n (%) |  |  | **< 0.001** |  |  | **< 0.001** | |  |  | **0.003** |
| G1 | 62 (11.5%) | 36 (6.7%) |  | 70 (12.9%) | 28 (5.2%) |  | | 64 (11.8%) | 34 (6.3%) |  |
| G2 | 70 (12.9%) | 50 (9.2%) |  | 78 (14.4%) | 42 (7.8%) |  | | 61 (11.3%) | 59 (10.9%) |  |
| G3 | 141 (26.1%) | 182 (33.6%) |  | 124 (22.9%) | 199 (36.8%) |  | | 148 (27.4%) | 175 (32.3%) |  |
| Overall survival （OS）, n (%) |  |  | **< 0.001** |  |  | 0.054 | |  |  | **0.009** |
| Survive | 245 (44.4%) | 213 (38.6%) |  | 238 (43.1%) | 220 (39.9%) |  | | 241 (43.7%) | 217 (39.3%) |  |
| Death | 31 (5.6%) | 63 (11.4%) |  | 38 (6.9%) | 56 (10.1%) |  | | 35 (6.3%) | 59 (10.7%) |  |
