# Supplementary material for: m6A‐related long noncoding RNAs predict prognosis and indicate therapeutic response in endometrial carcinoma
Source: J Clin Lab Anal. 2022 Dec 16;37(1):e24813. doi: 10.1002/jcla.24813 (PMC9833960; doi:10.1002/jcla.24813)
Supplement: Supplementary file 4 — Table S4. [file JCLA-37-e24813-s005.docx]

**Table S4 Construction of m6A LncRNA-associated ceRNA**

| **LncRNA** | **mRNA** | **miRNA** |
| --- | --- | --- |
| **CDKN2B-AS1** | **EPHA7** | hsa-miR-302d-3p,hsa-miR-302c-3p,hsa-miR-378d,hsa-let-7b-5p,hsa-miR-181c-5p,hsa-miR-520d-3p,hsa-miR-4429,hsa-miR-422a,hsa-let-7e-5p,hsa-miR-548o-3p,hsa-miR-320b,hsa-miR-199b-5p,hsa-miR-520b,hsa-miR-302a-3p,hsa-miR-98-5p,hsa-miR-378f,hsa-let-7f-5p,hsa-miR-4524b-5p,hsa-miR-181a-5p,hsa-let-7d-5p,hsa-miR-4500,hsa-miR-181b-5p,hsa-miR-302b-3p,hsa-miR-378e,hsa-miR-4458,hsa-miR-4524a-5p,hsa-miR-497-5p,hsa-miR-320a,hsa-miR-320c,hsa-miR-15b-5p,hsa-miR-320d,hsa-let-7i-5p,hsa-let-7c-5p,hsa-miR-6805-3p,hsa-miR-372-3p,hsa-miR-15a-5p,hsa-miR-378a-3p,hsa-miR-378h,hsa-miR-16-5p,hsa-miR-373-3p,hsa-miR-4262,hsa-miR-378i,hsa-let-7g-5p,hsa-miR-199a-5p,hsa-miR-424-5p,hsa-miR-6838-5p,hsa-miR-520a-3p,hsa-miR-5094,hsa-miR-5691,hsa-miR-520e,hsa-miR-378b,hsa-miR-378c,hsa-miR-3613-5p,hsa-miR-302e,hsa-miR-181d-5p,hsa-miR-195-5p,hsa-let-7a-5p,hsa-miR-1323,hsa-miR-330-3p,hsa-miR-520c-3p |
|  | **PIM1** | hsa-miR-542-3p |
|  | **AK9** | hsa-miR-181b-5p,hsa-miR-199a-5p,hsa-miR-4429,hsa-miR-181a-5p,hsa-miR-181c-5p,hsa-miR-181d-5p,hsa-miR-320d,hsa-miR-378c,hsa-miR-16-5p,hsa-miR-378d,hsa-miR-320a,hsa-miR-378a-3p,hsa-miR-195-5p,hsa-miR-199b-5p,hsa-miR-320c,hsa-miR-330-3p,hsa-miR-378b,hsa-miR-497-5p,hsa-miR-6838-5p,hsa-miR-514a-5p,hsa-miR-422a,hsa-miR-4262,hsa-miR-320b,hsa-miR-424-5p,hsa-miR-378i,hsa-miR-378f,hsa-miR-1323,hsa-miR-15b-5p,hsa-miR-15a-5p,hsa-miR-378h,hsa-miR-548o-3p,hsa-miR-378e |
|  | **ILK** | hsa-miR-542-3p |
|  | **TLK1** | hsa-let-7g-5p,hsa-miR-378i,hsa-miR-4458,hsa-miR-4524b-5p,hsa-let-7i-5p,hsa-miR-330-3p,hsa-let-7e-5p,hsa-miR-320a,hsa-miR-654-3p,hsa-miR-320b,hsa-let-7f-5p,hsa-let-7a-5p,hsa-miR-195-5p,hsa-miR-15a-5p,hsa-miR-378c,hsa-miR-320d,hsa-miR-324-5p,hsa-miR-378f,hsa-miR-199a-5p,hsa-let-7d-5p,hsa-miR-98-5p,hsa-miR-6838-5p,hsa-miR-424-5p,hsa-miR-378a-3p,hsa-miR-181d-5p,hsa-miR-122-5p,hsa-miR-4524a-5p,hsa-miR-5094,hsa-miR-320c,hsa-let-7c-5p,hsa-miR-181b-5p,hsa-miR-1323,hsa-miR-199b-5p,hsa-miR-4262,hsa-miR-4429,hsa-miR-378b,hsa-miR-181a-5p,hsa-miR-497-5p,hsa-miR-4500,hsa-miR-548o-3p,hsa-miR-378e,hsa-let-7b-5p,hsa-miR-378d,hsa-miR-3613-5p,hsa-miR-16-5p,hsa-miR-422a,hsa-miR-378h,hsa-miR-15b-5p,hsa-miR-181c-5p |
|  | **BIRC5** | hsa-miR-542-3p |
|  | **GPD2** | hsa-miR-4262,hsa-miR-302b-3p,hsa-miR-654-3p,hsa-miR-373-3p,hsa-miR-302c-3p,hsa-miR-15a-5p,hsa-miR-324-5p,hsa-miR-195-5p,hsa-miR-520d-3p,hsa-miR-15b-5p,hsa-miR-320a,hsa-miR-199a-5p,hsa-miR-302e,hsa-miR-6838-5p,hsa-miR-181c-5p,hsa-miR-1323,hsa-miR-520a-3p,hsa-miR-122-5p,hsa-miR-302a-3p,hsa-miR-6805-3p,hsa-miR-16-5p,hsa-miR-320b,hsa-miR-497-5p,hsa-miR-181a-5p,hsa-miR-5691,hsa-miR-302d-3p,hsa-miR-520b,hsa-miR-4524b-5p,hsa-miR-320d,hsa-miR-4429,hsa-miR-5094,hsa-miR-520e,hsa-miR-330-3p,hsa-miR-320c,hsa-miR-181b-5p,hsa-miR-3613-5p,hsa-miR-548o-3p,hsa-miR-372-3p,hsa-miR-520c-3p,hsa-miR-514a-5p,hsa-miR-4524a-5p,hsa-miR-181d-5p,hsa-miR-199b-5p,hsa-miR-424-5p |
| **YEATS2-AS1** | **CAMK2D** | hsa-miR-185-5p |
|  | **SCARB1** | hsa-miR-185-5p |
|  | **CAMK4** | hsa-miR-185-5p |
|  | **ITCH** | hsa-miR-214-5p |
|  | **RUNX1** | hsa-miR-378a-3p |
|  | **CDK6** | hsa-miR-214-5p,hsa-miR-378a-3p,hsa-miR-185-5p |
|  | **TCEAL1** | hsa-miR-370-3p |
|  | **ALOX12** | hsa-miR-185-5p |
|  | **ATR** | hsa-miR-185-5p |
|  | **BCL6** | hsa-miR-339-5p |
|  | **SP1** | hsa-miR-324-5p |
|  | **GNAI1** | hsa-miR-320c |
|  | **LCOR** | hsa-miR-615-3p |
|  | **MDM2** | hsa-miR-339-5p |
|  | **SRCIN1** | hsa-miR-873-5p |
|  | **IGF1R** | hsa-miR-185-5p |
|  | **NTRK3** | hsa-miR-185-5p |
|  | **KSR1** | hsa-miR-378a-3p |
| **SLC16A1-AS1** | **GLI2** | hsa-miR-218-5p |
|  | **CYLD** | hsa-miR-130b-3p |
|  | **HMOX1** | hsa-miR-218-5p |
|  | **NR3C1** | hsa-miR-130b-3p |
|  | **HOXB3** | hsa-miR-218-5p |
|  | **SMO** | hsa-miR-218-5p |
|  | **HMGB1** | hsa-miR-218-5p |
|  | **UVRAG** | hsa-miR-130b-3p |
|  | **E2F2** | hsa-miR-218-5p |
|  | **POU2F2** | hsa-miR-218-5p |
|  | **RSBN1** | hsa-miR-5688,hsa-miR-4782-3p,hsa-miR-3142,hsa-miR-411-5p,hsa-miR-1245b-5p,hsa-miR-224-3p,hsa-miR-495-3p,hsa-miR-219a-5p,hsa-miR-9-3p,hsa-miR-4637,hsa-miR-380-3p,hsa-miR-19b-3p,hsa-miR-126-5p,hsa-miR-134-5p,hsa-miR-3118,hsa-miR-580-3p,hsa-miR-522-3p,hsa-miR-6766-3p,hsa-miR-19a-3p,hsa-miR-582-3p,hsa-miR-499b-5p,hsa-miR-6509-3p |
