# Supplementary material for: m6A‐related long noncoding RNAs predict prognosis and indicate therapeutic response in endometrial carcinoma
Source: J Clin Lab Anal. 2022 Dec 16;37(1):e24813. doi: 10.1002/jcla.24813 (PMC9833960; doi:10.1002/jcla.24813)
Supplement: Supplementary file 5 — Table S5. [file JCLA-37-e24813-s002.docx]

**Table S5 Primer sequences for m6A-associated LncRNA**

| **Gene Name** | **Bidirectional primer sequences** | **Annealing temperature(℃)** | **Product length(bp)** |
| --- | --- | --- | --- |
| GAPDH | F:5’GGGAAACTGTGGCGTGAT3’  R:5’GAGTGGGTGTCGCTGTTGA3’ | 60 | 299 |
| CDKN2B-AS1 | F:5'GGGTTCAAGCATCACTGTTAGGT3’  R:5’ CCTCTGATGGTTTCTTTGGAGTT3’ | 60 | 118 |
| MIR924HG | F:5'ACCACCGAGTTGACAAAAGT3’  R:5’ GCTGCTGGAGGTTTACTTGA 3’ | 60 | 66 |
| YEATS2-AS1 | F:5'ATACACGTTCCTGTGGAGTG3’  R:5’ TGTCAGGGAGGTATGCTTGT 3’ | 60 | 126 |
